# Effective community-based interventions to prevent and control infectious diseases in urban informal settlements in low- and middle-income countries: a systematic review

**DOI:** 10.1186/s13643-024-02651-9

**Published:** 2024-10-04

**Authors:** Sohana Shafique, Dipika Shankar Bhattacharyya, Iffat Nowrin, Foyjunnaher Sultana, Md Rayhanul Islam, Goutam Kumar Dutta, Mariam Otmani del Barrio, Daniel D. Reidpath

**Affiliations:** 1 Health Systems and Population Studies Division (HSPSD), icddr,b, Dhaka, Bangladesh; 2 UNICEF/UNDP/World Bank/WHO Special Programme for Research and Training in Tropical Diseases (TDR), World Health Organization, Geneva, Switzerland

**Keywords:** Community-based intervention, Infectious diseases, Social determinants of health, Systematic review, Low- and middle-income countries

## Abstract

**Background:**

The impact of rapid urbanization taking place across the world is posing variegated challenges. Especially in terms of communicable disease, the risk is more concentrated in urban poor areas where basic amenities are inadequate. This systematic review synthesizes evidence on the effective community-based interventions (CBIs) aimed at preventing and controlling infectious diseases among the urban poor in low- and middle-income countries (LMICs).

**Methods:**

This systematic review was conducted following the PRISMA (Preferred Reporting Items for Systematic Reviews and Meta-Analysis) guidelines. A comprehensive search across five major databases was conducted to capture literature on CBIs published between 2011 and 2021. Scientific articles of any design that reported any type of CBIs effective in preventing and controlling infectious diseases (tuberculosis, diarrhea, typhoid, dengue, hepatitis B and C, influenza, and COVID-19) were included. Screening and selection of studies were done by two pairs of independent researchers using the predefined eligibility criteria. The risk of bias in included studies was assessed using the modified checklist outlined in the Cochrane Handbook for Systematic Reviews of Interventions and Effective Public Health Practice Project (EPHPP). Analysis of effective CBIs was guided by the conceptual framework for integrated CBIs for neglected tropical diseases (NTDs), and narrative synthesis was carried out. Geographical restrictions were limited to LMICs and papers published in English.

**Results:**

Out of 18,260 identified papers, 20 studies met the eligibility criteria and were included in this review. Community-based screening and socio-economic support, community-based vector control, behavior change communication, capacity building of the community health workers (CHWs), health education, and e- and m-health interventions were found as effective CBIs. Diversified CBIs were found to be effective for specific diseases, including tuberculosis (TB), diarrhea, dengue, influenza and ARI, and hepatitis B and C. Bundling of interventions were found to be effective against specific diseases. However, it was difficult to isolate the effectiveness of individual interventions within the bundle. The socio-cultural context was considered while designing and implementing these CBIs.

**Conclusion:**

The effectiveness of an intervention is inextricably linked to social context, stakeholder dimensions, and broader societal issues. System approach is recommended, emphasizing context-specific, multi-component interventions that address social determinants of health. Integrating these interventions with public health strategies and community involvement is crucial for sustainable outcomes. These findings can guide the design of future interventions for better prevention and control of communicable diseases in urban poor areas.

**Systematic review registration:**

PROSPERO CRD42021278689.

**Supplementary Information:**

The online version contains supplementary material available at 10.1186/s13643-024-02651-9.

## Background

Over the last century, urbanization has impacted the social and demographic characteristics of human beings around the world [1, 2]. Currently, half of the world’s population resides across urban areas, which is projected to rise to 70% by 2050 [3]. This urbanization is concentrated mainly in low- and middle-income countries (LMICs) and according to recent estimates, 90% of the urbanization takes place in Africa and Asia [4]. While the urban environment offers many opportunities and services, it concentrates health risks and introduces disproportionate hazards for more vulnerable groups, especially the poor living in informal settlements among whom food insecurity, inadequate housing, and limited social protection play a role in increasing the burden of disease [5, 6]. An urbanized place needs to have some specific characteristics to provide basic amenities and services to the urban people such as a municipal governance structure, infrastructural services, health services, and economic activity to support the urban population. However, across the LMICS, the rapid and unplanned urbanization in a changing climate is characterized by substandard housing with weak infrastructure, unemployment, and scarcity of basic services such as the supply of food, safe drinking water, adequate sanitation, hygiene, etc. Moreover, in the urban informal settlements, environmental exposure to pathogens, pollutants and chemicals, indoor and outdoor air pollution, inadequate food systems, and poor access to already inadequate health services pose a high risk for infectious diseases for urban dwellers [7, 8].

The social, economic, and environmental factors are the contributors to shape human behavior and influence their health and wellbeing for which they are referred to collectively as social and environmental determinants of health (SDOH) [9, 10]. The World Health Organization (WHO) defines the social determinants of health as a complete set of social and physical conditions where people live and work, including social, economic, demographic, environmental, and cultural factors, along with the health system [11]. Due to the suboptimal condition of the SDOH in urban informal settlements, there is an aggravated risk of emerging and persistent infectious diseases, particularly in the LMICs [12]. For example, the recent COVID-19 pandemic has affected people around the world, but due to high population density and poor housing infrastructure, the urban slums were disproportionately more vulnerable to virus exposure and infections [13]. This is further evident from a COVID-19 seroprevalence study conducted in Bangladesh, which suggested that the seroprevalence of COVID-19 IgG and/or IgM was 45% in the Dhaka city whereas it was 74% in the urban slums [14]. The urban poor living in informal settlements are vulnerable to the spread of infectious diseases, such as influenza, pneumonia, and tuberculosis [15]. Therefore, to inform the design of an effective intervention for the prevention and control of infectious diseases in urban informal settlements in LMICs, there is a need to recognize the heterogeneity of the living conditions of urban poor and require a broad multifaceted approach considering the SDOH [16, 17].

Across the LMICs, community-based interventions (CBIs) that put emphasis on grassroots participation from the community by enabling them in health-related decision-making became popular in the 1960s and early 1970s [18]. To date, the CBIs are found to be cost-effective and scalable approaches in terms of the prevention and management of infectious diseases [19–22]. However, the majority of current CBIs are mainly directed toward disease-specific programs that take a vertical approach [23]. The vertical approach refers to instances where a targeted health problem is sought to be solved through the application of a particular measure provided by single-purpose machinery [24]. A lot of these vertical approaches do not tend to include a lens for analyzing how power relations contribute to complex and multiple forms of health advantages and disadvantages. The intersectional approach is an analytical framework that focuses on how multiple dimensions of social inequalities interact and intersect with each other at different levels to co-construct unequal health outcomes at individual and population levels [24–26]. In addition, the Special Program for Research and Training in Tropical Diseases (TDR) (tropical disease research) has recently further defined an intersectional gender approach, by specifically encompassing a gender lens as the entry point for a deeper intersectional analysis [27]. The intersectional gender approach analyzes how gender power relations intersect with other social stratifies to affect people’s lives and create differences in needs and experiences, with an aim to inform policies, services, and programs for addressing those differences [27].

However, there is a dearth of knowledge on effective community-based interventions that take an intersectional gender approach to address the complex SDOH of infectious disease in urban poor settings in the LMICs. Considering the importance of gender roles, there is a need for evidence on how the design and implementation of the CBIs can be beneficial by taking an intersectional gender approach. In this regard, evidence from LMIC settings considering the context of urban informal settlements and lessons learned from the experiences of different countries will provide an opportunity to develop context-specific scalable interventions. The primary objective of this systematic review was to identify effective community-based interventions to prevent and control infectious diseases in urban informal settlements in LMICs. The secondary objective of this review was to identify factors influencing the implementation of community-based interventions using an intersectional gender lens.

## Methods

This systematic review has been conducted following the criteria of Preferred Reporting Items for Systematic Reviews and Meta-Analysis Protocols (PRISMA-P) [28]. This systematic review protocol was registered with PROSPERO (CRD42021278689).

### Expert consultation

Prior to developing the search strategy, an expert consultation was performed to refine the preliminary research questions of the review through the collation of feedback received from 20 stakeholders and public health experts in the arena of infectious diseases, urban health, and social determinants of health. A concept note containing two initially proposed review questions was shared with them and the research questions and methods were revised based on the feedback received.

### Eligibility criteria

This review included intervention studies defined by the Effective Practice and Organization of Care (EPOC) group [29]. The review included randomized controlled trials (RCT), non-randomized trials/quasi-experimental studies, cluster randomized trials, repeated measures studies, interrupted time series studies, and controlled before-after studies. Studies reporting any type of CBI in preventing and controlling infectious diseases or evaluating the effect of CBI on infectious diseases among the urban poor in LMICs. The selection of LMICs followed the World Bank classification, as it provides a recognized standard for identifying countries with limited resources where such interventions are most critically needed [30]. Studies only published in English were considered. The publication time of the review was limited to the period from 2011 to the end of September 2021. The year 2011 marked a significant milestone in the field of public health with the convening of the World Conference on SDOH. During this event, the “RIO Political Declaration” on SDOH signified a global commitment to addressing the social factors that profoundly impact individuals’ well-being and health outcomes [31]. Studies published as editorial, letters, opinions, brief communications, or short reports were excluded. The search strategy was developed based on the following population, intervention, comparison, and outcome (PICO) model.

### Population

The review included studies that were conducted in the settings among the urban poor in countries in Asia, Latin America, and sub-Saharan Africa. Studies that report on the residents of slums, informal settlements, or street dwellers in urban areas in the selected regions were included. There is great diversity in the definition of what constitutes the urban area. Urban areas can be categorized based on different indicators or a combination of them including population size, population density, the number of non-agricultural workers, infrastructure like roads and utilities, and the presence of education and healthcare services [5, 7]. However, in our systematic review, we did not limit our search based on any specific characteristic to define the urban areas. Since there is no unanimously accepted global definition of urban [7], we used search terms like “urban,” “peri-urban,” “city,” “municipality,” “urbanization,” and “township.” No restriction was placed in terms of sex, age, and ethnicity.

### Intervention

This review considered studies focusing on any type of community-based intervention or programs implemented by governments, NGOs, international organizations, and research organizations for the prevention and control of selected infectious diseases among the urban poor. We defined the “community-based intervention (CBI)” as an intervention that was designed to prevent infectious diseases at the population level. The CBI covered two broad definitions—interventions that were implemented at the community level and interventions that engaged or provided ownership to the community in decision-making during the design and delivery of the intervention [32].

### Comparison

No comparison was measured under this review.

#### Outcome

The incidence of the selected diseases was measured as the main outcome. In addition to it, morbidity and mortality due to those selected diseases were also measured.

### Data sources and searches

An updated and comprehensive search strategy was developed using the key terms and synonyms such as “urban poor” OR “poverty areas” OR “slum” OR “informal settlement” AND “prevention and control” OR “practice” OR “awareness” OR “community intervention” AND “COVID-19” OR “tuberculosis” OR “hepatitis” OR “diarrhea” OR “typhoid fever” OR “dengue” OR “influenzas” OR “pneumonia” AND “LMIC.” We have searched four major electronic databases (PubMed, Web of Science, Scopus, and Cochrane Library) and 3ie database of impact evaluations. The search through the electronic database was conducted on November 8, 2021. Several relevant articles have been reviewed and cross-checked to assess the comprehensiveness of the search strategy. According to our review question, while building the search strategies, we did not take the intersectional gender approach. Intersectionality was used to address the secondary objective.

### Study selection and data extraction

**Fig. 1:**
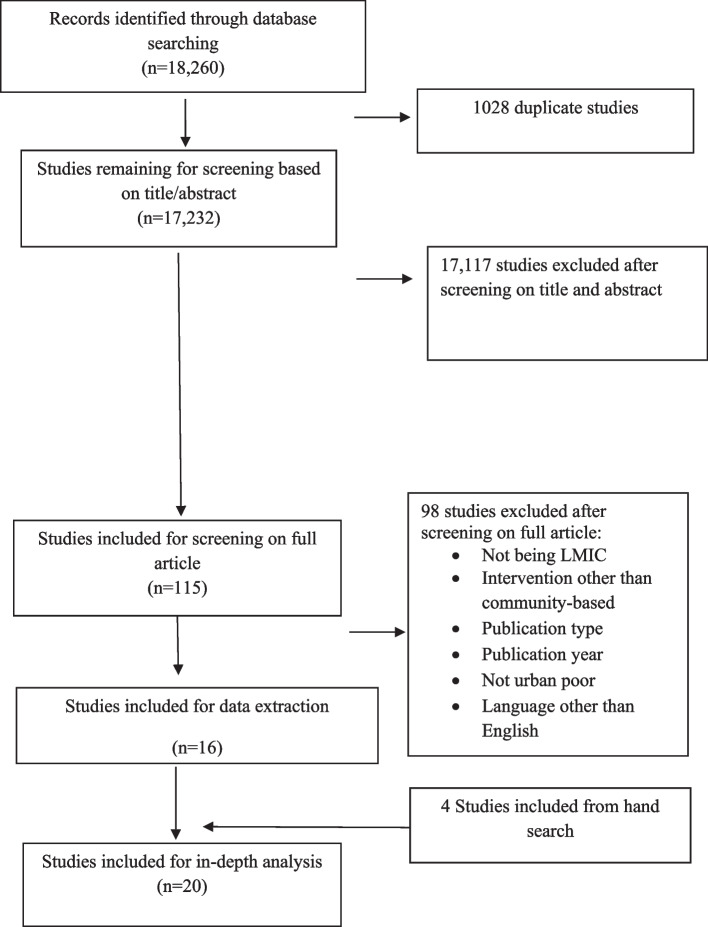
PRISMA flow diagram of study selection process

The articles retrieved from the initial search were exported in Rayyan QCRI software (online), and then, duplicates were removed and the studies were screened by title and abstract by using this software. Then, the studies were screened by full text and selected for data extraction following the inclusion and exclusion criteria. Four reviewers divided into two groups were involved in the screening process. Two reviewers from each group independently screened the included studies by title and abstract and accordingly full text. The lead reviewer was involved in resolving any kind of dispute. Since the systematic review was conducted following PRISMA guidelines, a flow diagram has been provided demonstrating the summary of all included and excluded articles (Fig. 1).

Two reviewers independently extracted data using a data extraction form containing some basic data that included the following criteria: author, publication year, publication type (e.g., original research), study design, country, intervention details, and outcome assessed. Before starting data extraction, pilot data extraction was done with two eligible studies to check the suitability and feasibility of the data extraction tool. At this stage of piloting, every reviewer participated in data extraction using the same studies to check the consistency and a similar understanding of data extraction. Any disagreement between the reviewers was resolved through discussion with the lead reviewer, and an opinion was taken from the review team if necessary.

### Quality assessment

Two pairs of reviewers independently assessed the risk of bias using a checklist. Any uncertainties and discrepancies were resolved by discussion, further review of the respective study, and consultations with a third reviewer, where necessary. The risk of bias assessment checklist was adopted from the criteria outlined in the Cochrane Handbook for Systematic Reviews of Interventions and Effective Public Health Practice Project (EPHPP) checklist [33]. In this review, “blinding” was not considered as a quality assessment criterion as blinding of participants or intervention implementers is rare in community-based intervention. The checklist followed in this review was also adopted and modified from the systematic review done by Hossain et al. [33]. Moreover, we categorized methodological components as “high/ “medium”/ “low” in terms of quality adopted from published systematic reviews [33]. The items of “random sequence generation” and “allocation concealment” were not considered in order to assess the quasi-experimental study and cohort study included in this review.

### Data analysis and synthesis

Studies reporting the effectiveness of any community-based intervention in preventing and controlling selected infectious diseases, such as tuberculosis, diarrhea, typhoid, dengue, hepatitis B and C, influenza, and COVID-19, were included. As the main outcome, data on the incidence and prevalence (proportion) of the selected infectious diseases were extracted. In addition, data on relative risk ratio (RRR), adjusted risk ratio (ARR), adjusted odds ratio (AOR), incidence rate, and mean difference of the outcomes were extracted. Community-based interventions that resulted in improved outcomes or behavior (e.g., decreased incidence and prevalence, hospitalization rate; improved immunization; increased knowledge and practice on handwashing) were defined as effective. Analysis of effective CBIs for the prevention and control of infectious diseases was guided by the conceptual framework for integrated community-based interventions for neglected tropical diseases (NTDs) [32]. Meta-analysis was not possible to conduct due to significant heterogeneity in intervention types, comparison groups, outcomes of interest, outcome measurement, and statistical analysis. Instead, a descriptive analysis of the study findings was done.

To address the secondary objective, a narrative synthesis was carried out to identify the facilitating factors for implementing the effective intervention in overcoming social, economic, and gender inequalities in the urban poor context. For this, we combined the SODH framework of the Commission on Social Determinants of Health (CSDH), World Health Organization (WHO) [10] with the WHO toolkit on “incorporating intersectional gender analysis into research on infectious diseases of poverty” [27] to describe intervention implementation strategy.

## Results

### Study selection

For this systematic review, a total of 18,260 published articles were selected and 1028 duplicate articles were removed, providing 17,232 titles and abstracts for review. After applying the inclusion and exclusion criteria, a total of 115 studies were considered for full-text screening. Among them, 98 articles were excluded and 16 were included. References were checked for all included articles and an additional four articles were included after citation tracking. Finally, a total of 20 articles were considered for the systematic review. Figure 1 provides a detailed “flow diagram” for the study selection process.

### Characteristics of included studies

**Fig. 2:**
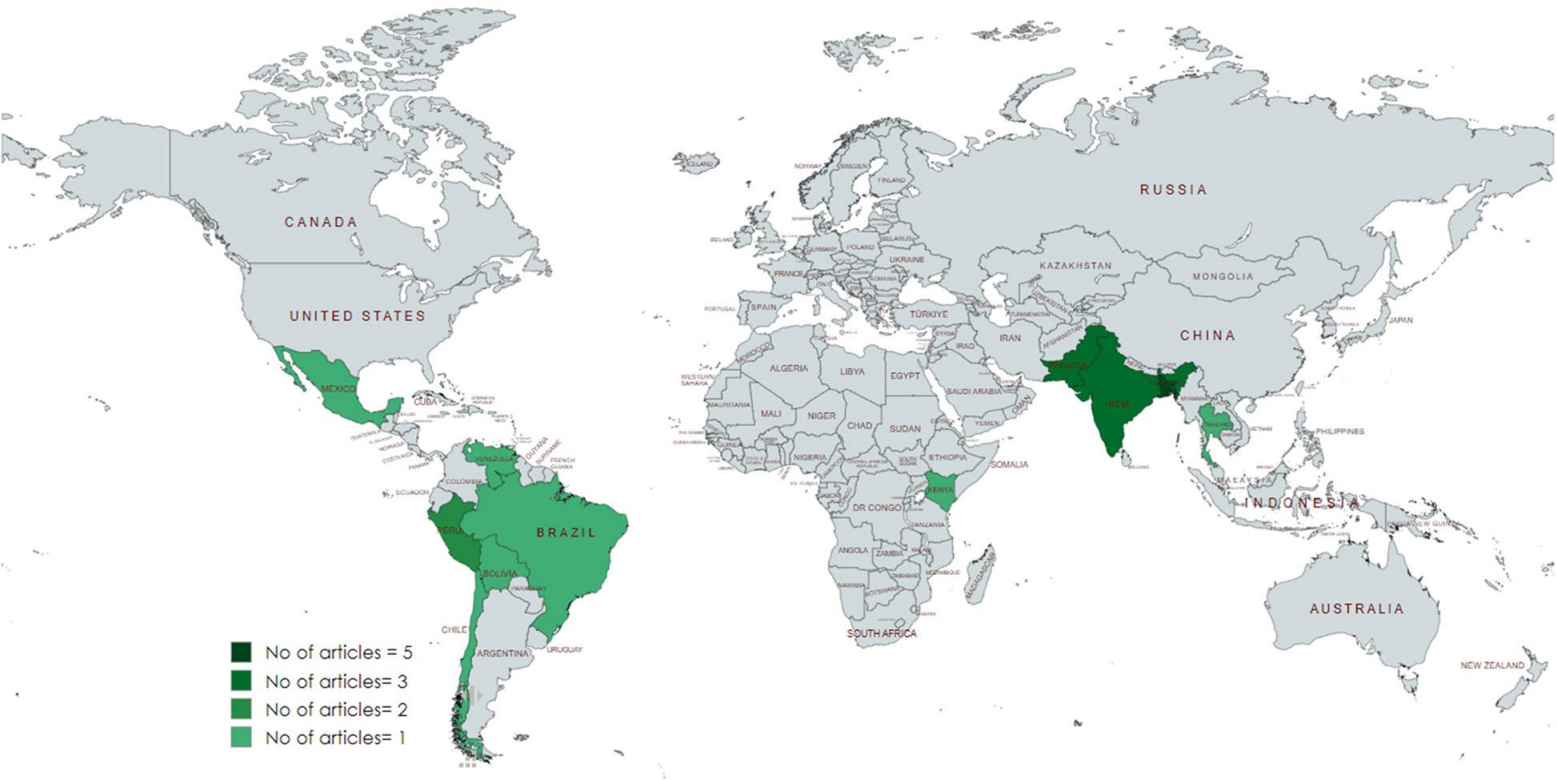
Geographical distribution of the included studies (*n* = 20)

The primary studies included in this review were sourced from 11 different countries and published in peer-reviewed journals between 2011 and 2021. The geographical distribution of the studies shows that Bangladesh was the most frequently represented country (*n* = 5) followed by Pakistan (*n* = 3) and India (*n* = 3) (Fig. 2). By regional distribution, South Asia was the common setting (12 studies out of 20). Regarding study design, most of the studies were RCT (*n* = 9) and CRT (*n* = 6) (Table 1). Few articles were quasi-experimental (*n* = 3) and cohort (*n* = 2), while two articles did not specify their study design. The predominant disease was tuberculosis (*n* = 7) followed by diarrhea (*n* = 6), dengue (*n* = 3), and influenza (*n* = 2). Other diseases such as hepatitis B and C, Haemophilus influenza, diarrhea, and ARIs have one article each (Table 1).

**Table 1:** Characteristics of the included studies by region, intervention type, disease, study design, and sample size

Characteristics	Detail characteristics	*n* (%)
**Region**	South Asia (Bangladesh, India, Pakistan)	11 (55)
	South America (Venezuela, Peru, Brazil, Bolivia)	5 (25)
	South-East Asia (Cambodia, South-East Asia)	2 (10)
	East Africa (Kenya)	1 (5)
	North America (Nicaragua)	1 (5)
**Intervention type**	WASH intervention	5 (25)
	Health education	4 (20)
	Community-based screening	4 (20)
	Socio-economic support	2 (10)
	Community-based vector control	2 (10)
	Behavior change intervention	1 (5)
	Social mobilization	1 (5)
	e- and m-health interventions	1 (5)
**Disease**	TB	7 (35)
	Diarrhea	6 (45)
	Dengue	3 (15)
	Influenza and ARI	3 (15)
	Hepatitis B and C	1 (5)
**Study design**	Randomized controlled trials	9 (45)
	Cluster randomized controlled trial	6 (30)
	Quasi-experimental	3 (15)
	Cohort study	1 (10)
	Pre-post study	1 (10)
**Sample size**	< 1000	4 (20)
	1000–3000	6 (30)
	>3000	8 (40)

### Quality of the review

Study quality and the risk of bias in included studies were assessed systematically following a checklist adopted and modified from the criteria outlined in the Cochrane Handbook for Systematic Reviews of Interventions and Effective Public Health Practice Project (EPHPP) checklist [33]. Robust methods were adopted to minimize error and bias. A comprehensive systematic search of major electronic databases to identify studies. In addition, references of included studies were checked. Randomized control trials and quasi-experimental trials were considered to capture all varieties of interventions. More than half of the included studies were of high quality (13 studies), 6 studies were of medium quality, and only one study was of low quality. However, the review has several limitations that potentially influenced its findings. Most of the interventions were complex, incorporating multiple components. It was difficult to indicate the effectiveness of any specific component in driving significant changes in the outcomes.

### Effective community-based interventions

A range of CBIs focusing on different diseases were found in our review. These include health education [34–37], socio-economic support [38, 39], behavior change communication [40], water, sanitation and hygiene (WASH) interventions [41–45], e- and m-health [46], social mobilization [47], community-based screening [48–51], and community-based vector control [52, 53]. This review identified multi-prong approaches and strategies to tackle specific diseases. In the subsequent section, we detailed the effective interventions identified from the review targeting selected infectious diseases (Table 2).

**Table 2:** Characteristics of the intervention studies included in the systematic review

**Author, year**	**Study design**		**Target population**	**Intervention components**	**Intervention focus**		**Results**
**Health education**							
Kusuma et al., 2016 [34]	Quasi-experimental		Slum population	1. Dissemination of health educational material (pamphlets, posters, banners, audio message recordings)	To increase knowledge and awareness on dengue		Significant increase in knowledge on the cause, symptom perception, and mosquito behaviour
Ong’ang’o et al., 2014 [35]	Retrospective cohort		TB patients receiving treatment from Public health facilities supported by the govt	1. Provision of health education by CHWs 2. Supervision of DOTS 3. Follow-up home visit by CHWs	To improve knowledge and awareness of TB treatment through CHWs among Intensive and continuous-phase patients		Increased adherence to treatment in the intervention arm who utilized CHWs (83%) vs control (64%); (OR)-2.65 (2.02-3.48)
Kaewchana et al, 012 [36]	Randomised control trial (RCT)		Household members over the age of 7 years residing with a child diagnosed with influenza.	1. Individual training and memorizing messages about hand washing technique 2. Provision of hand washing supplies 3. Hand washing education on influenza infection and benefits of hand washing	To improve the frequency and quality of hand washing and increase knowledge on influenza		Increased frequency of hand washing from 4.1 (SD = 2.7) to 5.6 (SD = 3.5) Improved quality score: 3.2 (SD=1.3) to 6.5 (SD-1.8).
Owais et al., 2011 [37]	RCT		All mothers living in the study areas and having a child ≤ 6 weeks old.	1. Distribution of pictorial cards by the trained CHWs to the mother containing messages regarding importance of vaccine, logistic information and the significance of retaining immunization cards for school admissions	To increase DPT and Hepatitis-B immunization coverage		DPT-3/Hepatitis B immunization rates in the intervention group were improved by 39% (unadjusted RR = 1.39; 95% CI: 1.07 - 1.82).
**Socio-economic support**							
Rocha et al, 2011 [38]	RCT		TB patients and their household contacts, Household contacts are defined as people spending >2 hours thrice weekly in the home with a TB patient during their illness.	1. Home visits to TB-affected households 2. Fortnightly community-mobilisation workshops 3. Psychological counselling, principally for depression and substance abuse 4. Poverty reduction activities, food and cash transfers 5. Income generation activities through microenterprise, microcredits and vocational training	To improve uptake of TB prevention, diagnosis and treatment services		Improved Household contact TB screening: (from 82% to 96%) Increased successful TB treatment completion (from 91% to 97%); *P* < 0.0001 Increased uptake of rapid MDR-TB testing (from 67% to 92%); *P* < 0.0001 Improved preventive therapy completion rate (from 27% to 87%); *P* < 0.0001
Wingfield et al., 2017 [39]	RCT		TB Patients under treatment and their household contacts	1. Education on tuberculosis transmission, treatment and preventive therapy through household visits and participatory community meetings 2. Formation of TB support group/club 3. Conditional cash transfer for treatment	To improve knowledge on TB treatment, reduce the stigma of TB and empower the TB patients		Improved preventive therapy completion: OR=1.9 (95% CI: 1.1 - 3.2) Increased preventive therapy initiation in contacts younger than 5 years (aOR: 2.2; 95% CI: 1.1–4.2) and in the poorest tercile (aOR: 2.2; 95% CI: 1.1–4.1)
**Behaviour change interventions**							
Biswas et al., 2019 [40]	Cluster RCT	Children of 5-10 aged going to primary school		1. Placement of hand sanitizer in each classroom and outside toilets at each school 2. Hand and respiratory hygiene education		To encourage children to use the hand sanitizer at five key times during the day	Decreased incidence of influenza-like illness (ILI) episode (at least one episode= 15% and more than one episode: 3%) Decreased incidence of influenza virus infection in intervention schools: 3.0/1,000 student-weeks) Vs control schools: 6.2/1,000 student-weeks
Qadri et al., 2015 [41]	RCT	Non-pregnant residents of high risk in terms of socioeconomic status and sanitation and hygiene.		1. Establishment of a free handwashing station in a convenient place of household compounds 2. Distribution of free soapy water bottle and sachet of soap 3. Provision of chlorine dispenser beside each drinking water station		To promote handwashing and home treatment of drinking water with liquid chlorine	Improved OCV coverage:Vaccination-only group: 65% Vaccination plus behaviour change group: 66%
Najnin et al., 2017 [42]	RCT	General population of selected community		1. Interpersonal counselling on hand-washing and water treatment aided by support print materials 2. Provide hardware enabling hand-washing and water treatment		To improve behaviour related to water purification and handwashing behaviour	Reduced hospitalization rate due to severe diarrhea: Vaccine-only vs vaccine-plus BCC= 4.7/1000 person-years (95% CI: 4.1–5.6) vs 4.1/1000 person-years (95% CI: 3.4–5.0); control= 4.7/1000 person-years (95% CI: 3.9–5.8)
**Water Sanitation and Hygiene (WASH) interventions**							
Lindquist [43]	RCT	under five children		1. Provision of free household-level hollow fiber filter and 2. Behavior change communication (BCC) to increase hand washing		To reduce the diarrheal disease in children less than 5 years of age	Reduction in diarrhea prevalence ratio: Filter arm: 0.21; [95% CI] = 0.15–0.30) Filter and WASH BCC arm: 0.27 (95% CI = 0.22–0.34)
Nicholson et al., 2014 [44]	Clustered RCT	Household living without a water supply or sanitation, household income of less than US$60/month		1. Provision of free soap 2. Five years olds children were taught through songs, poems and stories during classrooms visits. 3. Establishment of a ‘Good Mums’ club with the help of the mother enlisted through home visits. 4. Establishment of social norms for child and mother by using the fear of contamination. 5. “Best mum competition”- a network of mothers and parents meeting to boost morale 6. Remuneration to all target children		Behaviour change to educate, motivate and reward children for hand washing and sanitation	Under-5 children Diarrhoea: RRR-21.3 ARI- 19.9 Number of days school absences: Child illness- 26.7
Pickering et al., 2019 [45]	Clustered RCT	Children younger than 5 years and corresponding households who drank water from a designated water point		1. Establishment of a water storage tank compatible with a dosing device for water purification		To achieve high uptake of in-line chlorinated drinking water to reduce diarrhoea	Reduced hospital visits for diarrhoea: Intervention vs Control: 7.5% vs 10% Gastrointestinal illness: Intervention vs Control: 3.7% vs 4%,
**e- and m-Health interventions**							
Kazi et al., 2018 [46]	RCT	Children less than 2 weeks of age, whose parent or guardian or at least one person in thehousehold has a valid mobile phone connection		SMS reminders for routine immunization by sending text message saying “Child name’ is due for 6-week vaccination immediately take your child to the nearest EPI center.”		To improve vaccination coverage	Significant increase in Pentavalent 1 (DPT-Hep-B-Hib) immunization coverage: (at 6 weeks) Intervention arm: 96.0% (86/90) vs Control arm: 86.4% (102/118) (*p*<0.03)
**Social/Community mobilization**							
khan et al, 2013 [47]	RCT	High-risk population for cholera		1. Distribution of cards, sending cell phone messages related to OCV for social mobilization 2. Banners presentation at vaccination sites to create awareness 3. Carrying out additional vaccination sessions for garments workers 4. Mop-up activities for the second dose		To increase OCV vaccine coverage	Improved vaccine coverage in children: 81% Significant Coverage in females compared to males (77% vs. 66%, *P* < 0.001) Increased rate in receiving first and second doses of vaccine: (82%) and (72%) respectively Decreased dropout rate from the first to the second dose: 13%.
**Community-based screening**							
Potty et al., 2021 [49]	Cohort study	slum population		1. Follow-up visits with counselling and contact screening 1. Provision of social scheme at continuation phase		To improve knowledge and uptake of TB treatment and testing services	The annualized casedetection rate increased from 5.5 to 52.0/100 000 in Bengaluru and increased up to 118.9 /100 000 persons in Hyderabad Increased treatment success rate (87.1%) in the first cohort vs 91.3% in the last cohort
Lorent et al; 2014 [50]	Cohort study	Communities living in informal temporary or unstructured settlements with a presumed high prevalence of undiagnosed TB and/or restricted access to TB services.		1. Screening ofall available household members and symptomatic cases of TB by door-to-door visits. 2. Facilitation of TB screening by home collection and transport to sputum specimen to laboratories. 3. Referral of positive cases to local health facility through quick diagnosis. 4. Active tracking and encouraging TB patients who dropped out or interrupted treatment through offering convenient treatment modality (community- or ambulatory-DOTS)		To enhance community screening for active TB case detection and treatment initiation	Increased rate in treatment Initiation: 95%, Increased successful treatment outcome: 81%;
Fatima et al., 2014 [48]	Quasi experimental	General population		1. Screening of TB among persons reported > 2 weeks cough by on site trained microscopist for LED-FM examination of sputum 2. Referral system for diagnosed TB cases. 2. Provision of treatment by the provincial TB control programme under DOTS-based system		To increase no of TB detention	Tuberculosis detection increased Detected in camp- 12.7% Detected by GPs- 18.2%
Soares et al., 2013 [51]	Pre-post	General population People with high notification rates		1. An active case finding campaign to increase case detection 2. A standardized door-to-door symptom screening and spot sputum collection for symptomatic individuals. 3. Supervision of treatment and doses by trained CHWs 4. Provision of monthly medical consultation. 5. Implementation of educational activities 6. Social networking for anti-TB treatment.		To strengthen local TB control by improving access to health services, increasing community awareness and reducing the duration of infectiousness in the community.	Significant improvement in TB Treatment outcomes: Increased treatment success rates: 67.6% vs. 83.2%, (*P* <0.001) Reduction in default rates: 17.8% vs. 5.5%, *P* <0.001).
**Community based vector control**							
Vanlerberghe et al., 2011 [52]	Clustered RCT	General population		1. Delivery of insecticide treated (IT) curtains and IT jar		To improve the coverage of ITM	Significant reduction in dengue Incidence Rate Ratio = 0.98; 95%CI: 0.97–0.99
Andersson et al., 2015 [53]	Clustered RCT	Children aged 3-9 years		1. Community discussion of baseline results 2. Chemical-free prevention of mosquito reproduction		To enhance knowledge and awareness on dengue prevention and control measures	Decreased rates in self reporting dengue illness in intervention compared to control group: (5.7% vs 7.1%) House index improved: houses infested with larvae or pupae/houses: Control (Mean): 19.6% Intervention (Mean): 13.6%

### Health education

Across the reviewed studies, providing health education was identified as the most prevalent community-based intervention [34–37]. In different studies, health education was reported as an effective intervention to address different types of diseases rather than any particular disease. Kusuma et al. (2016) conducted a quasi-experimental study in the slum population in Delhi, India, to assess the effect of health education on dengue prevention [34]. Health education on dengue transmission and preventive measures was provided through the dissemination of materials such as pamphlets, posters, banners, and audio message recordings. Findings showed a significant increase in awareness that dengue was caused by mosquitoes (20%, *p* < 0.0001). The use of mosquito repellents/coils and wearing clothes covering full and bed net was increased after receiving this educational intervention by ~ 20%, 11%, and 6% respectively (*p* < 0.0001). Health education along with counseling by trained community health workers (CHWs) also improved treatment adherence (15%) in contrast to the control group. Utilization of CHW’s most effective in urban setup [OR − 2.65 (95% CI 2.02 − 3.48; *p* < 0.001)], combining the use of CHWs and treatment adherence had a strong positive association [OR − 8.02 (95% CI 5.43 − 11.88, *p* < 0.001)] [3].

Kaewchana et al. conducted an RCT where health education was provided to control influenza and its consequences as well [36]. This educational intervention was repeatedly provided to the household individual on handwashing technique and conveying messages such as “Why to wash,” “when to wash,” “how to wash,” and “how handwashing is linked to influenza transmission.” This intervention was able to increase the frequency of handwashing in the intervention group (*p* < 0.001). In addition, findings showed that in comparison to pre-education and 90 days post-education in the intervention group, the frequency was increased from 4.1 (SD = 2.7) to 5.6 (SD = 3.5). Intervention in handwashing techniques made significant improvement in the duration of handwashing (*p* < 0.001). It was self-reported by the participants that the frequency of their handwashing increased after using their hands to cover their mouth and nose when coughing, sneezing, and blowing their nose and after touching any other surfaces that were presumed secretion-contaminated (*p* < 0.001). These findings were identified as the strengths of educational approaches [36]. Health education intervention implemented for mothers of children less than 6 months to increase their knowledge about immunization (through pictorial cards containing messages about the importance of immunization, retaining vaccine card, and logistic information by CHWs) showed a significant improvement of 39% (adjusted RR = 1.39; 95% CI 1.06─1.81) in DPT-3/hepatitis B vaccine completion rates in the intervention group [37].

### Socio-economic support

Targeting the TB patients and their households, a socio-economic support was implemented in Peru through several components to tackle TB [38, 39]. To enhance the uptake of TB care and prevention services, home visits, community mobilization workshops, and psychosocial support were integrated within the national TB control program to reduce the burden. Considering poverty as another barrier to the success of TB controlling program poverty-reduction interventions (food and cash transfers, microcredits, microloans, etc.) were also delivered, which engaged 77% of the participants in these interventions. The socio-economic intervention showed a marked increase in TB screening (from 82 to 96%), successful TB treatment completion (from 91 to 97%), and completion of preventive therapy (from 27 to 87%; all *p* < 0.0001) [38].

Wingfield et al. [39] reported social and economic support through regular household visits, participatory community meetings, education on TB transmission, treatment and preventive therapy, formation of a TB support club, and conditional cash transfer which yielded a treatment success rate of 64% for the intervention group compared to 53% for the control group. Furthermore, the cure rate was higher in the intervention group at 53%, as opposed to 37% in the control group. The rate of preventive therapy completion was 20% in the intervention group, compared to 12% in the control group, with an OR of 1.9 (95% CI 1.1–3.2). The intervention notably increased the initiation of preventive therapy in contacts younger than 5 years (aOR 2.2; 95% CI 1.1–4.2) and among the poorest tertile (aOR 2.2; 95% CI 1.1–4.1), highlighting its effectiveness in these specific subgroups. This multifaceted social support was designed to inform, empower, and reduce TB stigma within the community, while economic support was directly targeted at individual patients [39].

### Behavior change interventions

Behavior change intervention was applied in a cluster randomized control trial conducted among children of 24 primary schools in Dhaka, Bangladesh, to prevent influenza, as they are commonly linked to influenza transmission. To promote handwashing practice in low-resource settings where water is scares, hand sanitizer was provided instead of water and soap to intervention schools which were regularly filled by field staff during the intervention period. Besides, hand and respiratory hygiene education was delivered through trained selected teachers regarding proper ways to cover during coughing and sneezing and the use of hand sanitizer at five key times during the day. Each of the enrolled students was provided a plastic ruler containing messages on handwashing with soap and respiratory hygiene etiquette. In addition, a video clip previously developed by icddr,b scientists based on respiratory hygiene practice was delivered during behavior change communication sessions to the students. At the end of the intervention, coughing and sneezing in the open air among the students at intervention schools decreased to 37% (DID =  − 63%; 95% CI = 98%, − 27%). Around 18% lower incidence of influenza-like illness (ILI) per 1000 student-weeks was identified among the students of intervention school than that of control school adjusted incidence rate ratio (AIRR): 0.8, 95% CI 0.5─1.3, *p* value < 0.05)]. Around 53% of lower incidence of laboratory-confirmed influenza per 1000 student-weeks was identified among students of intervention school than that of control school [incidence rate ratio (IRR): 0.5, 95% CI = 0.3, 0.8; *p* value < 0.01)] [40]. In another study, behavior change intervention along with vaccination was applied and compared with other only vaccinated and control group (no intervention) to assess the effectiveness of OCV vaccine in reducing the incidence of severe dehydrating cholera among high-risk people of age above 1 year and except pregnant women during 2 years after vaccination. In this cluster randomized study, a BCC was applied along with vaccination to encourage handwashing and treatment of drinking water with chlorine by trained CHWs to promote the use of a liquid chlorine-based treatment for household drinking water, each drinking water station included a chlorine dispenser. Overall protective effectiveness was 37% (95% CI lower bound 18%; *p* < 0.01) in the vaccination group and 45% (95% CI lower bound 24%; *p* < 0.001) in the vaccination and BCC group [41].

### Water sanitation and hygiene (WASH) interventions

Four out of 20 studies [42–45] reported WASH interventions aimed at reducing the incidence of diarrheal diseases and acute respiratory infections (ARI) in different settings. In Bangladesh, oral cholera vaccine (OCV) and WASH interventions provided protection against severe dehydrating cholera in the vaccination plus BCC group (45%; 95% CI 13–55; *p* < 0.001) [42]. Najnin et al. [42] assessed the impact of handwashing and water purification on oral cholera vaccination (OCV) in Bangladesh and found a reduction in diarrhea-associated hospitalization rates in the vaccine-plus-BCC group (4.1/1000 person-years; 95% CI 3.4–5.0) compared to the vaccine-only (4.7/1000 person-years; 95% CI 4.1–5.6), and control groups over a period of 2 years (4.7/1000 person-years; 95% CI 3.9–5.8) [42]. Lindquist et al. [43] evaluated the efficacy of household-level hollow fiber filters and BCC on WASH. A significant reduction in diarrheal disease in children under five using the filters was shown by diarrheal prevalence ratios of 0.21 (95% CI 0.15–0.30) for the filter arm and 0.27 (95% CI 0.22–0.34) for the filter and WASH BCC arm [43]. Effects of a handwashing intervention on health outcomes and school absenteeism in Indian urban communities showed a relative risk reduction in diarrhea RRR-21.3 and ARIs (ARI-19.9) among children with a reduction in the number of days of school absences [44]. Pickering et al. [45] evaluated the effect of a water storage tank compatible with dosing devices at shared water points in urban Bangladesh which reported a reduction in diarrheal prevalence and hospital visits for gastrointestinal illnesses among children under five (control − 4%, intervention − 3.7%) [45].

### e- and m-health intervention

Kazi et al. [46] conducted a study in Pakistan to examine the effectiveness of SMS reminders on the uptake of routine immunizations in urban squatter settlements. The study targeted infants under 2 weeks of age, whose caregivers had mobile phone access and could read SMS text messages. The intervention group showed a higher rate of immunization coverage for the first dose of the pentavalent vaccine (DPT-Hep-B-Hib) at 6 weeks compared to the control group (intervention arm, 96.0% vs. control arm, 86.4%; *p* < 0.05) [46].

### Social mobilization

In 2013, Khan et al. conducted a RCT in the high-risk cholera-endemic urban areas of Dhaka, Bangladesh, to assess the feasibility and impact of a large OCV program aimed at reducing cholera incidence. The program involved social mobilization strategies, including interpersonal communication by field workers, advocacy meetings, and targeted mobile messages to ensure high vaccine coverage among the susceptible urban population. To further boost coverage, mop-up activities were conducted, involving house-to-house visits targeting those who missed the second dose. The study found that vaccine coverage was 81% among children, and coverage among females was significantly higher than in males (77% vs. 66%, *p* < 0.001) [47].

### Community-based screening

A quasi-experimental study was undertaken to increase TB case detection by adopting an integrated intervention which included arranging chest camps for active case detection at the clinics of private non-NTP general practitioners (GPs) and using a light-emitting diode (LED) microscope with fluorescence microscopy. Local GPs received 3 days of training regarding diagnosis, recording, and reporting of TB in the provincial TB control program and were encouraged to refer TB presumptive cases to temporary laboratories in a nearby GP clinic. Promotional activities were assumed prior to the chest camps such as announcing through loudspeakers about the camps and free general medicine including displaying posters and banners in Urdu and in the local Sindhi language. To attract the local community, health fairs were arranged that included street theatre, fun shows, and stalls. This integrated intervention is evident that the proportion of smear-positive results was significantly higher among those from engaged private providers than among those referred from camps (OR 1.54, 95%CI 1.42–1.66). During the project, the total number of smear-positive TB notifications increased over the intervention period from 5158 to 8275 [48].

Another study was conducted among the slum population of two cities, Hyderabad and Bengaluru, India, through USAID-funded Tuberculosis Health Action Learning Initiative (THALI) to support them in gaining access to TB services. To increase awareness among the slum population, THALI trained 112 CHWs and placed them in urban slums to visit and conduct activities in the slums fortnightly. They referred symptomatic TB cases to the nearest center for sputum testing and also visited households with positive cases. They also supported TB patients and families with counseling, contact screening, monitoring treatment adherence, weight follow-up during visits, and social scheme linkages. Their counseling also covered relevant behavior change (smoking and alcohol consumption) and referring the TB patients to a doctor for management of adverse effects or side effects management and co-morbidities. These community-based activities through the CHWs showed an increase in TB detection rate in Bengaluru from 5.5 to 52.0 per 100,000 during the period, while in Hyderabad, it was 35.4 initially and increased up to 118.9 per 100,000 persons. The treatment success rate was 87.1% through the intervention. Weight (OR 1.60, *p* < 0.05), the total number of follow-up visits (OR 10.73, *p* < 0.001), TB awareness counseling (OR 2.75, *p* < 0.001), adherence counseling (OR 3.34, *p* < 0.001), nutritional counseling/support (OR 2.43, *p* < 0.001), and family level counseling (OR 1.90, *p* < 0.05) were intriguing factors for the successful treatment outcome [48].

In Cambodia, a study assessed the feasibility and effectiveness of community-based active case finding (ACF) for TB in disadvantaged urban areas which reported a high initiation of treatment (95%), a successful treatment outcome (81%), a cure rate (69%), and a completion rate (12%) over a period of 51 days [50]. Rocinha, Brazil’s largest urban slum, implemented capacity building of the laypersons as CHWs to supervise TB treatment, launching a campaign to find out active cases of TB, home visits for screening symptomatic individuals, and educational activities to enhance TB control. This multi-prong intervention resulted in an increased treatment success rate (from 67.6 to 83.2%). Furthermore, the TB case rate declined by an average of 39 cases per 100,000 population every 6 months in the post-intervention period [51].

### Community-based vector control

In Venezuela, a community-based vector control intervention was implemented to prevent and control dengue in urban informal settings [52, 53]. A CRT was conducted to evaluate the effectiveness of long-lasting insecticide-treated materials (ITMs), such as curtains and jars, for controlling *Aedes aegypti*, the primary vector for dengue. The results showed a significant impact, with an incidence rate ratio of 0.98, suggesting a slight reduction in dengue incidence due to the intervention [52].

The Camino Verde (Green Way), a pesticide-free evidence-based community mobilization, was added to the conventional dengue control program in the intervention sites to test whether it enhances effectiveness in dengue prevention in Nicaragua and Mexico. In this cluster, randomized control trial intervention sites followed a protocol to engage communities through a variety of events based on local vector reservoirs and community resources like puppet shows and basketball tournaments, clean-up campaigns focused on unoccupied and public premises, and introduction of fish into water storage containers for 1 year. These intervention sites participated in a community discussion of baseline evidence engaging the community leaders which helped to motivate community involvement during and beyond the study. Communities opted for a series of activities to raise awareness and share basic knowledge on the mosquito life cycle and how to interrupt it through volunteer visits at households and schools. This multi-country community-based study showed community mobilization to be an effective intervention for dengue vector control as household evidence of recent dengue virus infection among 3–9-year-old children was reduced (relative risk reduction (RRR) − 29.5, (95% CI − 3.8, − 55.3), *p* < 0.05), past self-reported dengue illness decreases (RRR) − 24.7, 95% CI − 1.8, − 51.2), *p* < 0.05), house infested with larvae or pupae (RRR − 44.1, 95% CI − 13.6, − 74.7), *p* < 0.001), containers with larvae or pupae (RRR 36.7, 95% CI − 24.5, − 44.8), *p* < 0.001), and the number of pupae (RRR 51.7, 95% CI − 36.2, − 76.1), *p* < 0.001) [40].

### Effectiveness of intervention for specific infectious diseases

This systematic review finds different types of community-based interventions for specific diseases, e.g., TB, dengue, diarrhea, influenza, and ARI. In this section, we have reported disease-wise intervention and their effectiveness (Table 3).

**Table 3:** Summary of the effective community-based intervention for prevention and control of infectious diseases in LMICs

Disease	Intervention settings	Intervention	Intervention components	Effectiveness
Tuberculosis	Household, neighborhood, and community	Health education	1. Provision of health education by CHWs [34]	Increased adherence to treatment [34]
			2. Supervision of DOTs [34] 3. Follow-up home visit by CHWs [34] [37] [38]	
		Socio-economic support	1. Income generation activities through microenterprise, microcredits, and vocational training [37] 2. Fortnightly community-mobilization workshops [37] 3. Psychological counseling, principally for depression, and substance abuse [37] 4. Poverty reduction activities, food, and cash transfers [37] [38] 5. Formation of TB support group/club [38]	Improved household contact TB screening [37] Increased successful TB treatment completion [37] Increased uptake of rapid MDR-TB testing [37] Improved preventive therapy completion rate [37] Increased preventive therapy initiation [37]
		Community-based screening	1. Follow-up visits with counseling and contact screening [48] 2. Provision of social scheme at communication phase [48] 3. Screening of all available household members and symptomatic cases of TB by door-to-door visits [49] 4. Facilitation of TB screening by home collection and transport of sputum specimens to laboratories [49] 5. Active tracking and encouraging TB patients who dropped out and interrupted treatment through offering convenient treatment modalities [49] 6. Screening of TB among persons reported > 2 weeks cough by on-site trained microscopist for LED-FM examination of sputum [47] 7. Referral system for diagnosed TB cases [47] 8. Provision of treatment by the provincial TB control program under DOTS based system [47] 9. An active case-finding campaign to increase case detection [50] 10. A standardized door-to-door symptom screening and spot sputum collection for symptomatic individuals [50] 11. Supervision of treatment and doses by trained CHWs [50] 12. Provision of monthly medical consultation [50] 13. Implementation of educational activities [50] Social networking for anti-TB treatment [50]	Increased case detection rate [47, 48] Increased treatment success rate [48, 49] Increased rate of treatment initiation [49] Significant improvement in TB treatment outcome [50]
Diarrhea	Household, neighborhood, community, school	Behavior changes interventions	1. Behavior change communication (BCC) to increase handwashing [42] 2. Distribution of free soapy water bottles and sachet of soap [40] 3. interpersonal counseling on handwashing and water treatment aided by support print materials [41] 4. Establishment of social norms for child and mother by using the fear of contamination [43]	Reduction in diarrhea prevalence [42] Improved OCV coverage [40] Reduced hospitalization rate due to diarrhea [41]
		Water sanitation and hygiene (WASH) intervention	1. Establishment of a free handwashing station in a convenient place of household compounds [40] 2. Provision of free household-level hollow fiber filter [42] 3. Establishment of a water storage tank compatible with a dosing device for water purification [44]	Improved OCV coverage [40] Reduction in diarrhea prevalence [42] Reduced hospital visits for diarrhea [44]
Dengue	Open place, neighborhood, household	Health education	1. Dissemination of health educational material (pamphlets, posters, banners, audio message recordings) [33]	Significant increase in knowledge on the cause, symptom perception, and mosquito behavior [33]
		Community-based vector control	1. Delivery of insecticide-treated (IT) curtains and IT jar [51] 2. Community discussion of baseline results [52] 3. Chemical-free prevention of mosquito reproduction [52]	Significant reduction in dengue incidence rate ratio [51] Decreased rates in self-reporting dengue illness [52]
Influenza and ARI	School, household	Health education	1. Handwashing education on influenza infection and benefits of handwashing [35] 2. Hand and respiratory hygiene education [39] 3. Provision of handwashing supplies [35] 4. Individual training and memorizing messages about handwashing techniques [35]	Increased frequency of handwashing [35]
		Behavior changes interventions	1. Placement of hand sanitizer in each classroom and outside toilets at each school [39]	Decreased incidence of influenza virus infection [39]
Hepatitis B and C	Community	Health education	1. Distribution of pictorial cards by the trained CHWs to the mother (regarding the vaccine, logistic information, and the significance of the immunization card) [36]	Hepatitis B immunization rates were improved [36]
		e- and m-health interventions	1. SMS reminders for routine immunization by sending text [45]	Significant increase in immunization coverage [45]

### Tuberculosis

Table 3 outlines various interventions for TB and their components along with their effectiveness. For health education, the interventions included health education by community health workers (CHWs), supervision of directly observed treatment (DOTs), and follow-up home visits, leading to increased adherence to treatment [35, 36, 39]. Socio-economic support involved income generation activities, community mobilization workshops, psychological counseling, poverty reduction activities, and forming TB support groups, which resulted in improved household contact TB screening, successful TB treatment completion, rapid MDR-TB testing uptake, and increased preventive therapy initiation [38, 39]. Community-based screening included follow-up visits with counseling, social scheme provision during communication phases, household and symptomatic case screening through door-to-door visits, and facilitating TB screening by home collection and transport, resulting in increased case detection, treatment success, and treatment initiation rates [48–50].

### Diarrhea

The interventions for diarrhea focus on BCC and WASH. Behavior change interventions include BCC for increased handwashing, distribution of soapy water and soap, interpersonal counseling on handwashing and water treatment, and establishing social norms for hygiene, resulting in reduced diarrhea prevalence, improved (OCV) coverage, and decreased hospitalization rates [41–44]. WASH interventions included setting up free handwashing stations, providing household-level hollow fiber filters, and establishing water storage tanks with dosing devices for water purification, leading to improved OCV coverage, reduced diarrhea prevalence, and fewer hospital visits for diarrhea [41, 43, 45] (Table 3).

### Dengue

The same table shows that health education and community-based vector control were effective interventions for the prevention and control of dengue. Health education involves disseminating educational materials such as pamphlets, posters, banners, and audio messages, leading to a significant increase in knowledge about dengue causes, symptom perception, and mosquito behavior [34]. Community-based vector control included delivering insecticide-treated (IT) curtains and jars, community discussions of baseline results, and chemical-free prevention of mosquito reproduction contributed towards a significant reduction in the dengue incidence rate ratio and decreased self-reporting of dengue illness [52, 53].

### Influenza and Acute Respiratory Infections (ARI)

One of the most effective interventions to combat influenza and ARI was health education including education on handwashing respiratory hygiene and individual training and demonstration on handwashing techniques. These interventions resulted in increasing the frequency of handwashing [36, 40]. Interventions on institutional changes that facilitated behavior change, e.g., providing handwashing supplies, specifically placing hand sanitizer in classrooms and toilets effectively decreased the incidence of influenza infection [40].

### Hepatitis B and C

Two interventions for improving hepatitis B and C immunization rates. The first intervention involved health education using pictorial cards distributed by trained CHWs to mothers. This intervention aimed at providing information on the vaccine, logistic information, and the significance of the immunization card. The second intervention utilized electronic and mobile health (e- and m-health) interventions, e.g., SMS reminders for routine immunization to increase immunization coverage. Both interventions showed positive results in improving hepatitis B and C immunization rates [37, 46].

Various community-based interventions, such as health education, socio-economic support, and community-based vector control, were effective in addressing specific diseases like TB, diarrhea, dengue, influenza, and hepatitis B and C in urban poor settings. The effectiveness of these interventions often involved increased adherence to treatment, improved disease prevention behaviors, and higher immunization rates. Interventions that addressed social determinants and were tailored to the local context showed effectiveness in terms of disease outcomes.

### Social determinants of infectious diseases and effectiveness of community-based Interventions (CBIs) through a gender lens

In this review, we have found that SODH can significantly influence the intervention delivery and outcome. Different strategies were implemented to overcome those intersecting social stratifiers to make the intervention program successful. To further investigate the interventions of included studies, we analyzed the outcomes with an intersectional gender lens, focusing on how gender intersects with other social stratifiers in the intervention under analysis and how such interventions address gender intersecting inequities.

This review identified facilitating factors related to intervention implementation, such as context-specific intervention design [35–37, 42, 45, 47, 48, 52], mass vaccination campaign [41, 47], mobile phone ownership and acceptability of receiving SMS [46], and a strong history of community engagement [53].

As evident in this review, TB tends to concentrate in poor and marginalized communities where gender further intersects with other social stratifiers (age, income, employment, migration status, geographic location) and interacts with the wider process of social and structural systems that shape their disease experience that include social forces, economy, and education system [39, 41]. Rocha et al. [38] have shown that overcrowded living conditions intersect with poverty, making them more vulnerable to TB [38]. Moreover, the unstable employment of this vulnerable population and their experience of stigma have further shaped their access to and utilization of health services. So, integrated socio-economic and bio-medical interventions were considered. The project facilitated empowerment activities, including education, workshops, and a mothers’ pooled childcare cooperative to help women contribute to household incomes. To address poverty activities such as microcredit loans and microenterprise activities, food and cash transfers were undertaken. Vocational training including raising animals and home-based manufacturing was provided by local organizations. Psychological support was also provided for these marginalized people facing stigmatization and depression so that they could have better access to health services [38]. Wingfield et al. have shown that socio-economic intervention through conditional cash transfer was effective in increasing access to TB treatment [39]. Other innovative strategies were reported by different authors [39, 42, 48, 50] such as door-to-door screening for active TB in poor urban settlements to enhance their access to health services. To identify the poorest and most hard-to-reach, community leaders were involved in the intervention delivery [39].

Gender, as a social determinant of health and a relational construct of power, was addressed by authors as a barrier to achieving intervention outcomes in the prevention and control of infectious diseases [41, 44, 47]. Behavior change intervention along with OCV was adopted in a high-risk cholera-prone urban setting where over-crowded poor living conditions intersect with unsafe water use and poor hygiene practices. To increase access to information, cell phone messages and banners were displayed at vaccination sites including messages related to diarrhea presented by trained volunteers. Liquid chlorine-based treatment was promoted for drinking water in every household compound. To increase vaccine coverage, outreach vaccination sites were established. As a large number of participants were employed in ready-made garments (RMG) and other industries, holidays and early morning mobile sessions were scheduled to reach them. As the complete vaccination coverage was more in females, the author argued for future vaccination strategies for this adult male group [41].

Acute respiratory infection tends to develop from poor living conditions, low-income, overcrowded housing conditions without a safe water supply and sanitation. An intervention was targeted for mothers and children to reduce episodes of illness from diarrhea and ARI. To involve mothers, a “good mum” club was established to encourage their children to handwashing. Health education was provided through promoters through home and school visits. To encourage handwashing, free soap was distributed by field workers. To motivate handwashing practice, the children were regularly rewarded [44]. In another study, intervention was designed by analyzing how gender power relations intersect with other social determinants of health in creating differences in needs and experience [37]. To prevent and control hepatitis B, mothers with poor income and low literacy rates from urban settings was targeted to improve immunization coverage by educating them [37].

Some of the factors affecting the sustainability and scalability of interventions were mainstreaming of the community health workers, strong community engagement, operational integration with local administration, and continuous communication and interaction between governmental agencies and communities.

## Discussion

This review reported an overview of the effective interventions to prevent and control infectious diseases in urban informal settlements in LMICs. The use of SDOH and gender lens provided a perspective where the macro-level context (social and economic structure, culture) needs to be considered in implementing the interventions at the community level. In addition, the nature of interventions and the process of engaging the community have also been identified that may help in designing future interventions in this area.

Across the included articles it was observed that the CBIs have the potential to substantially prevent and control the infectious diseases among the urban poor communities. Previous evidence from across the globe is also congruent with these findings [54]. A study conducted by Kidane et al. [55] in Ethiopia revealed that child mortality caused by holoendemic malaria can significantly be reduced by providing peer training intervention to the mothers. The review also identified the importance of shared leadership and proper engagement of the community from the initial stage of the intervention.

The major strength of this review was this was one of the first systematic reviews that attempted to identify the effective community-based interventions that were implemented in urban poor areas of LMICs. The review adopted robust methods so that errors and biases can be minimized. All the major electronic databases and key websites were systematically searched in a comprehensive manner to identify and include relevant studies. An additional search was conducted among the included studies for checking references to include relevant studies. We included RCT and quasi-experimental trials to capture varieties of interventions provided for prevention and control of communicable diseases in urban poor settings. Study quality was systematically assessed following a standard quality assessment checklist as per the Cochrane Handbook for Systematic Reviews of Interventions [32]. Nearly half of the included studies were of medium to high quality. However, it was difficult to pinpoint a single-intervention component in the package making a significant change in outcome. This was because the interventions taken were complex and designed with multi-component variations in social settings.

In view of the evidence from this review, it was difficult to say whether a single social determinant of health was more important than others across different settings. Based on the context, different SDOHs can play different roles. Therefore, the intervention components need to be tailor-made in line with the context. Evidence pointed out that interventions were most likely to be effective when designed with a package of components. Contextualized intervention design, community engagement, stakeholder participation in all stages, use of gender-responsive and transformative approaches, investment in infrastructure development, and providing support services were some of the factors that made the interventions effective.

This review highlights TB interventions, including health education, socio-economic support, and community-based screening. These efforts enhanced treatment adherence, leading to higher treatment success and preventive therapy initiation. A study highlighted the role of public–private partnership in TB control in Zambia, demonstrating that health education and socio-economic support through community engagement significantly improved treatment adherence and outcomes, similar to the findings of the present review [56]. The interventions for diarrhea focused on BCC and WASH, enhancing hygiene practices and water purification, resulting in reduced diarrhea prevalence, improved OCV coverage, and decreased hospitalization rates. Intensive handwashing promotion in high-risk communities in Pakistan significantly reduced the incidence of childhood diarrhea. Similar to the WASH interventions highlighted in the summary of the present review, one study in the LMIC setting underscores the effectiveness of BCC and hygiene practices in lowering diarrhea prevalence, demonstrating the impact of hygiene promotion on public health outcomes [57]. Similar to our findings, one study in Colombia found that CBIs significantly increased public awareness and led to a marked decrease in dengue case detection [58]. One study found that hand hygiene education and the use of hand sanitizers significantly decreased the incidence of influenza and ARI, similar to this review’s findings [59]. One meta-analysis found that e-health tools significantly enhanced immunization rates by improving hepatitis B and C immunization through health education and digital reminders [60].

This review provided an overview of the effectiveness of a wide range of community-based interventions with different types of approaches for a number of communicable diseases in urban poor areas in LMICs. It was observed that the articles that reported statistically non-significant outcomes were linked with short duration of intervention, small sample size, and large attrition effect. Given the complexity of community-based intervention, short-term intervention might not be efficient in reducing and controlling communicable diseases in an instant manner. Therefore, the evidence could be suggestive of an intervention model to try out in other contexts. Despite these limitations, some studies showed some promising results in terms of changing hospitalization rates, and disease incidence rates as well as changed attitudes, and behavior. Besides from a gender perspective, some of the studies also take an inclusive approach to include women. The review did not find any stand-alone community-based intervention approach to prevent and control communicable diseases.

This review and its analysis drew conclusions from multiple studies that include randomized and quasi-experimental designs. Besides the included studies have diversified context in terms of setting and population. Most of the studies came from Asia and Latin America with a few from the African region. The review shows that more large-scale, high-quality research is essential to provide further evidence about the effect of certain community-based interventions.

## Conclusion

Community-based intervention requires multisectoral and multi-level interactions and engagement across a wide range of stakeholders. The effectiveness of any intervention thus does not depend only on methodological approaches but also on the context and the social determinants of health. From this perspective, this review is suggestive of co-creating the intervention packages with community members so that community ownership and community leadership are ensured in the intervention implementation. Future research should also focus on appropriate outcome measures and include process indicators so that implementation challenges can be identified for future scaling up. Preventing and controlling infectious diseases in urban informal settlements in LMICs necessitates a comprehensive and system-thinking approach with the participation of all relevant stakeholders so that the long-term sustainability of these interventions can be ensured.

## Supplementary Information

Supplementary Material 1.

## Data Availability

The datasets used and/or analyzed during the current study are available from the corresponding author upon reasonable request.

## Abbreviations

ARI: Acute respiratory infections
BCC: Behavior change communication
CASP: Critical appraisal skills programe
CBI: Community-based intervention
CHW: Community health workers
DOTS: Directly observed treatment, short course
GP: General practitioner
icddr,b: International Center for Diarrhoeal Disease Research, Bangladesh
ILI: Influenza-like illness
INGO: International non-governmental organization
LMICs: Low- and middle-income countries
NGO: Non-governmental organization
OCV: Oral cholera vaccine
RCT: Randomized control trial
RRR: Relative risk reduction
SDOH: Social determinants of health
TB: Tuberculosis
TDR: Tropical disease research
WASH: Water, sanitation and hygiene
WHO-TDR: World Health Organization (WHO), Special Program for Research and Training in Tropical Diseases (TDR)
