# Supplementary material for: Effective community-based interventions to prevent and control infectious diseases in urban informal settlements in low- and middle-income countries: a systematic review
Source: Syst Rev. 2024 Oct 4;13:253. doi: 10.1186/s13643-024-02651-9 (PMC11451040; doi:10.1186/s13643-024-02651-9)
Supplement: Supplementary file 1 — Supplementary Material 1. [file 13643_2024_2651_MOESM1_ESM.docx]

**Supplementary Material 1**

**Search Strategy for PubMed**

Population

Poor term:

"poverty areas"[MeSH Terms] OR ("poverty"[All Fields] AND "areas"[All Fields]) OR "poverty areas"[All Fields] OR "slum"[All Fields] OR "informal settlement"[All Fields] OR "informal settlements"[All Fields] OR ("poverty"[MeSH Terms] OR "poverty"[All Fields] OR "poor"[All Fields]) OR "disadvantage*"[All Fields]

LMIC+Urban+poor

((("emerging country" [all fields] OR "emerging countries" [all fields] OR "emerging nation"[all fields] OR "emerging nations" [all fields] OR "emerging population"[all fields] OR "emerging populations"[all fields] OR "developing country"[tiab] OR "developing countries"[tiab] OR "developing nation"[tiab] OR "developing nations"[tiab] OR "developing population"[tiab] OR "developing populations"[tiab] OR "developing world"[tiab] OR "less developed country"[tiab] OR "less developed countries"[tiab] OR "less developed nation"[tiab] OR "less developed nations"[tiab] OR "less developed population"[tiab] OR "less developed populations"[tiab] OR "less developed world"[tiab] OR "lesser developed country"[tiab] OR "lesser developed countries"[tiab] OR "lesser developed nation"[tiab] OR "lesser developed nations"[tiab] OR "lesser developed population"[tiab] OR "lesser developed populations"[tiab] OR "lesser developed world"[tiab] OR "under developed country"[tiab] OR "under developed countries"[tiab] OR "under developed nation"[tiab] OR "under developed nations"[tiab] OR "under developed population"[tiab] OR "under developed populations"[tiab] OR "under developed world"[tiab] OR "underdeveloped country"[tiab] OR "underdeveloped countries"[tiab] OR "underdeveloped nation"[tiab] OR "underdeveloped nations"[tiab] OR "underdeveloped population"[tiab] OR "underdeveloped populations"[tiab] OR "underdeveloped world"[tiab] OR "middle income country"[tiab] OR "middle income countries"[tiab] OR "middle income nation"[tiab] OR "middle income nations"[tiab] OR "middle income population"[tiab] OR "middle income populations"[tiab] OR "low income country"[tiab] OR "low income countries"[tiab] OR "low income nation"[tiab] OR "low income nations"[tiab] OR "low income population"[tiab] OR "low income populations"[tiab] OR "lower income country"[tiab] OR "lower income countries"[tiab] OR "lower income nation"[tiab] OR "lower income nations"[tiab] OR "lower income population"[tiab] OR "lower income populations"[tiab] OR "underserved country"[tiab] OR "underserved countries"[tiab] OR "underserved nation"[tiab] OR "underserved nations"[tiab] OR "underserved population"[tiab] OR "underserved populations"[tiab] OR"underserved world"[tiab] OR "under served country"[tiab] OR "under served countries"[tiab] OR "under served nation"[tiab] OR "under served nations"[tiab] OR "under served population"[tiab] OR "under served populations"[tiab] OR "under served world"[tiab] OR "deprived country"[tiab] OR "deprived countries"[tiab] OR "deprived nation"[tiab] OR "deprived nations"[tiab] OR "deprived population"[tiab] OR "deprived populations"[tiab] OR "deprived world"[tiab] OR "poor country"[tiab] OR "poor countries"[tiab] OR "poor nation"[tiab] OR "poor nations"[tiab] OR "poor population"[tiab] OR "poor populations"[tiab] OR "poor world"[tiab] OR "poorer country"[tiab] OR "poorer countries"[tiab] OR "poorer nation"[tiab] OR "poorer nations"[tiab] OR "poorer population"[tiab] OR "poorer populations"[tiab] OR "poorer world"[tiab] OR "developing economy"[tiab] OR "developing economies"[tiab] OR "less developed economy"[tiab] OR "less developed economies"[tiab] OR "lesser developed economy"[tiab] OR "lesser developed economies"[tiab] OR "under developed economy"[tiab] OR "under developed economies"[tiab] OR "underdeveloped economy"[tiab] OR "underdeveloped economies"[tiab] OR "middle income economy"[tiab] OR "middle income economies"[tiab] OR "low income economy"[tiab] OR "low income economies"[tiab] OR "lower income economy"[tiab] OR "lower income economies"[tiab] OR "low gdp"[tiab] OR "low gnp"[tiab] OR "low gross domestic"[tiab] OR "low gross national"[tiab] OR "lower gdp"[tiab] OR "lower gnp"[tiab] OR "lower gross domestic"[tiab] OR "lower gross national"[tiab] OR lmic[tiab] OR lmics[tiab] OR "third world"[tiab] OR "lami country"[tiab] OR "lami countries"[tiab] OR "transitional country"[tiab] OR "transitional countries"[tiab] OR Africa[tiab] OR Asia[tiab] OR Caribbean[tiab] OR West Indies[tiab] OR South America[tiab] OR Latin America[tiab] OR Central America[tiab] OR "Atlantic Islands"[tiab] OR "Commonwealth of Independent States"[tiab] OR "Pacific Islands"[tiab] OR "Indian Ocean Islands"[tiab] OR Afghanistan[tiab] OR Albania[tiab] OR Algeria[tiab] OR Angola[tiab] OR Antigua[tiab] OR Barbuda[tiab] OR Argentina[tiab] OR OR Aruba[tiab] OR Azerbaijan[tiab] OR Bangladesh[tiab] OR Barbados[tiab] OR Benin[tiab] OR Byelarus[tiab] OR Byelorussian[tiab] OR Belarus[tiab] OR Belorussian[tiab] OR Belorussia[tiab] OR Belize[tiab] OR Bhutan[tiab] OR Bolivia[tiab] OR Bosnia[tiab] OR Herzegovina[tiab] OR Hercegovina[tiab] OR Botswana[tiab] OR Brasil[tiab] OR Brazil[tiab] OR Bulgaria[tiab] OR Burkina Faso[tiab] OR Burkina Fasso[tiab] OR Upper Volta[tiab] OR Burundi[tiab] OR Urundi[tiab] OR Cambodia[tiab] OR Khmer Republic[tiab] OR Kampuchea[tiab] OR Cameroon[tiab] OR Cameroons[tiab] OR Cameron[tiab] OR Cape Verde[tiab] OR Central African Republic[tiab] OR Chad[tiab] OR China[tiab] OR Colombia[tiab] OR Comoros[tiab] OR Comoro Islands[tiab] OR Comores[tiab] OR Congo[tiab] OR Zaire[tiab] OR Costa Rica[tiab] OR Cote d'Ivoire[tiab] OR Ivory Coast[tiab] OR Cuba[tiab] OR Cyprus[tiab] OR Czechoslovakia[tiab] OR "Czech Republic" [tiab] OR Slovakia[tiab] OR Slovak Republic[tiab] OR Dominica[tiab] OR Dominican Republic[tiab] OR East Timor[tiab] OR East Timur[tiab] OR Timor Leste[tiab] OR Ecuador[tiab] OR \ OR El Salvador[tiab] OR Eritrea[tiab] OR Estonia[tiab] OR Ethiopia[tiab] OR Fiji[tiab] OR Gabon[tiab] OR Gabonese Republic[tiab] OR Gambia[tiab] OR Gaza[tiab] OR Georgia Republic[tiab] OR Georgian Republic[tiab] OR Ghana[tiab] OR Gold Coast[tiab] OR Greece[tiab] OR Grenada[tiab] OR Guatemala[tiab] OR Guinea[tiab] OR Guam[tiab] OR Guiana[tiab] OR Guyana[tiab] OR Haiti[tiab] OR Honduras[tiab] OR Hungary[tiab] OR India[tiab] OR Maldives[tiab] OR Indonesia[tiab] OR Jamaica[tiab] OR Kazakhstan[tiab] OR Kazakh[tiab] OR Kenya[tiab] OR Kiribati[tiab] OR Korea[tiab] OR Kosovo[tiab] OR Kyrgyzstan[tiab] OR Kirghizia[tiab] OR Kyrgyz Republic[tiab] OR Kirghiz[tiab] OR Kirgizstan[tiab] OR "Lao PDR"[tiab] OR Laos[tiab] OR Latvia[tiab] OR Lebanon[tiab] OR Lesotho[tiab] OR Basutoland[tiab] OR Liberia[tiab] OR Lithuania[tiab]OR Macedonia[tiab] OR Madagascar[tiab] OR Malagasy Republic[tiab] OR Malaysia[tiab] OR Malaya[tiab] OR Malay[tiab] OR Sabah[tiab] OR Sarawak[tiab] OR Malawi[tiab] OR Nyasaland[tiab] OR Mali[tiab] OR Marshall Islands[tiab] OR Mauritania[tiab] OR Mauritius[tiab] OR Agalega Islands[tiab] OR "Melanesia"[tiab] OR Mexico[tiab] OR Micronesia[tiab] OR Middle East[tiab] OR Moldova[tiab] OR Moldovia[tiab] OR Moldovian[tiab] OR Mongolia[tiab] OR Montenegro[tiab] OR Mozambique[tiab] OR Myanmar[tiab] OR Myanma[tiab] OR Burma[tiab] OR Namibia[tiab] OR Nepal[tiab] OR Netherlands Antilles[tiab] OR New Caledonia[tiab] OR Nicaragua[tiab] OR Niger[tiab] OR Nigeria[tiab] OR Northern Mariana Islands[tiab] OR OR Pakistan[tiab] OR Palau[tiab] OR Palestine[tiab] OR Panama[tiab] OR Paraguay[tiab] OR Peru[tiab] OR Philippines[tiab] OR Philipines[tiab] OR Phillipines[tiab] OR Phillippines[tiab] OR Poland[tiab] OR Portugal[tiab] OR Puerto Rico[tiab] OR Russia[tiab] OR Russian[tiab] OR Rwanda[tiab] OR Ruanda[tiab] OR Saint Kitts[tiab] OR St Kitts[tiab] OR Nevis[tiab] OR Saint Lucia[tiab] OR St Lucia[tiab] OR Saint Vincent[tiab] OR St Vincent[tiab] OR Grenadines[tiab] OR Samoa[tiab] OR Samoan Islands[tiab] OR Navigator Island[tiab] OR Navigator Islands[tiab] OR Sao Tome[tiab] OR Senegal[tiab] OR Serbia[tiab] OR Montenegro[tiab] OR Seychelles[tiab] OR Sierra Leone[tiab] OR Slovenia[tiab] OR Sri Lanka[tiab] OR Ceylon[tiab] OR Solomon Islands[tiab] OR Somalia[tiab] OR Sudan[tiab] OR Suriname[tiab] OR Surinam[tiab] OR Swaziland[tiab] OR Tajikistan[tiab] OR Tadzhikistan[tiab] OR Tadjikistan[tiab] OR Tadzhik[tiab] OR Tanzania[tiab] OR Thailand[tiab] OR Togo[tiab] OR Togolese Republic[tiab] OR Tonga[tiab] OR Trinidad[tiab] OR Tobago[tiab] OR Turkey[tiab] OR Turkmenistan[tiab] OR Turkmen[tiab] OR Tuvalu[tiab] OR Uganda[tiab] OR Ukraine[tiab] OR Uruguay[tiab] OR USSR[tiab] OR Soviet Union[tiab] OR Union of Soviet Socialist Republics[tiab] OR Uzbekistan[tiab] OR Uzbek OR Vanuatu[tiab] OR New Hebrides[tiab] OR Venezuela[tiab] OR Vietnam[tiab] OR Viet Nam[tiab] OR Zambia[tiab] OR Zimbabwe[tiab] OR Rhodesia[tiab] OR Developing Countries[Mesh] OR Africa[Mesh:NoExp] OR Africa South of the Sahara[Mesh:NoExp] OR Africa, Central[Mesh:NoExp] OR Africa, Eastern[Mesh:NoExp] OR Africa, Southern[Mesh:NoExp] OR Africa, Western[Mesh:NoExp] OR Asia[Mesh:NoExp] OR Asia, Central[Mesh:NoExp] OR Asia, Southeastern[Mesh:NoExp] OR Asia, Western[Mesh:NoExp] OR Caribbean Region[Mesh:NoExp] OR West Indies[Mesh:NoExp] OR South America[Mesh:NoExp] OR Latin America[Mesh:NoExp] OR Central America[Mesh:NoExp] OR "Atlantic Islands"[Mesh:NoExp] OR "Commonwealth of Independent States"[Mesh:NoExp] OR "Pacific Islands"[Mesh:NoExp] OR "Indian Ocean Islands"[Mesh:NoExp] OR Afghanistan[Mesh] OR Albania[Mesh] OR Algeria[Mesh] OR American Samoa[Mesh] OR Angola[Mesh] OR "Antigua and Barbuda"[Mesh] OR Argentina[Mesh] OR Azerbaijan[Mesh] OR Bahrain[Mesh] OR "Baltic States"[Mesh] OR Bangladesh[Mesh] OR Barbados[Mesh] OR Benin[Mesh] OR "Republic of Belarus"[Mesh] OR Belize[Mesh] OR Bhutan[Mesh] OR Bolivia[Mesh] OR Bosnia-Herzegovina[Mesh] OR Botswana[Mesh] OR Brazil[Mesh] OR Bulgaria[Mesh] OR Burkina Faso[Mesh] OR Burundi[Mesh] OR Cambodia[Mesh] OR Cameroon[Mesh] OR Cape Verde[Mesh] OR Central African Republic[Mesh] OR Chad[Mesh] OR Chile[Mesh] OR China[Mesh] OR Colombia[Mesh] OR Comoros[Mesh] OR Congo[Mesh] OR Costa Rica[Mesh] OR Cote d'Ivoire[Mesh] OR Croatia[Mesh] OR Cuba[Mesh] OR Cyprus[Mesh] OR Czechoslovakia[Mesh] OR Czech Republic[Mesh] OR Slovakia[Mesh] OR "Democratic Republic of the Congo"[Mesh] OR "Democratic People's Republic of Korea"[Mesh] OR Dominica[Mesh] OR Dominican Republic[Mesh] OR East Timor[Mesh] OR Ecuador[Mesh] OR El Salvador[Mesh] OR Eritrea[Mesh] OR Estonia[Mesh] OR Ethiopia[Mesh] OR "Equatorial Guinea"[Mesh] OR Fiji[Mesh] OR "French Guiana"[Mesh] OR Gabon[Mesh] OR Gambia[Mesh] OR "Georgia (Republic)"[Mesh] OR Ghana[Mesh] OR Greece[Mesh] OR Grenada[Mesh] OR Guatemala[Mesh] OR Guinea[Mesh] OR Guinea-Bissau[Mesh] OR Guam[Mesh] OR Guyana[Mesh] OR Haiti[Mesh] OR Honduras[Mesh] OR Hungary[Mesh] OR "Independent State of Samoa"[Mesh] OR India[Mesh] OR Indonesia[Mesh] OR Jamaica[Mesh] OR Kazakhstan[Mesh] OR Kenya[Mesh] OR Korea[Mesh] OR Kyrgyzstan[Mesh] OR Laos[Mesh] OR Latvia[Mesh] OR Lebanon[Mesh] OR Lesotho[Mesh] OR Liberia[Mesh] OR Lithuania[Mesh] OR "Macedonia (Republic)"[Mesh] OR Madagascar[Mesh] OR Malawi[Mesh] OR Malaysia[Mesh] OR Mali[Mesh] OR Mauritania[Mesh] OR Mauritius[Mesh] OR "Melanesia"[Mesh] OR Mexico[Mesh] OR Micronesia[Mesh] OR Middle East[Mesh:NoExp] ORMoldova[Mesh] OR Mongolia[Mesh] OR Montenegro[Mesh] OR Mozambique[Mesh] OR Myanmar[Mesh] OR Namibia[Mesh] OR Nepal[Mesh] OR Netherlands Antilles[Mesh] OR New Caledonia[Mesh] OR Nicaragua[Mesh] OR Niger[Mesh] OR Nigeria[Mesh] OR Pakistan[Mesh] OR Palau[Mesh] OR Panama[Mesh] OR Papua New Guinea[Mesh] OR Paraguay[Mesh] OR Peru[Mesh] OR Philippines[Mesh] OR Poland[Mesh] OR Portugal[Mesh] OR Puerto Rico[Mesh] OR "Republic of Korea"[Mesh] OR Romania[Mesh] OR Russia[Mesh] OR "Russia (Pre-1917)"[Mesh] OR Rwanda[Mesh] OR "Saint Kitts and Nevis"[Mesh] OR Saint Lucia[Mesh] OR "Saint Vincent and the Grenadines"[Mesh] OR Samoa[Mesh] OR Saudi Arabia[Mesh] OR Senegal[Mesh] OR Serbia[Mesh] OR Montenegro[Mesh] OR Seychelles[Mesh] OR Sierra Leone[Mesh] OR Slovenia[Mesh] OR Sri Lanka[Mesh] OR Somalia[Mesh] OR South Africa[Mesh] OR Sudan[Mesh] OR Suriname[Mesh] OR Swaziland[Mesh] OR Syria[Mesh] OR Tajikistan[Mesh] OR Tanzania[Mesh] OR Thailand[Mesh] OR Togo[Mesh] OR Tonga[Mesh] OR "Trinidad and Tobago"[Mesh] OR Tunisia[Mesh] OR Turkey[Mesh] OR Turkmenistan[Mesh] OR Uganda[Mesh] OR Ukraine[Mesh] OR Uruguay[Mesh] OR USSR[Mesh] OR Uzbekistan[Mesh] OR Vanuatu[Mesh] OR Venezuela[Mesh] OR Vietnam[Mesh] OR Yemen[Mesh] OR Yugoslavia[Mesh] OR Zambia[Mesh] OR Zimbabwe[Mesh] OR "Southern African Development Community" [all fields] OR "East African Community"[all fields] OR "West African Health Organisation"[all fields] OR "Sub Saharan Africa "[all fields] OR "SubSaharan Africa "[all fields])) OR (urban)) OR (((((slum) OR ("informal settlement")) OR ("informal settlements")) OR (poor)) OR (disadvantage*))

Intervention

"prevent*"[All Fields] OR ("controling"[All Fields] OR "controllability"[All Fields] OR "controllable"[All Fields] OR "controllably"[All Fields] OR "controller"[All Fields] OR "controller s"[All Fields] OR "controllers"[All Fields] OR "controlling"[All Fields] OR "controls"[All Fields] OR "prevention and control"[MeSH Subheading] OR ("prevention"[All Fields] AND "control"[All Fields]) OR "prevention and control"[All Fields] OR "control"[All Fields] OR "control groups"[MeSH Terms] OR ("control"[All Fields] AND "groups"[All Fields]) OR "control groups"[All Fields]) OR "communit*"[All Fields] OR ("knowledge"[MeSH Terms] OR "knowledge"[All Fields] OR "knowledge s"[All Fields] OR "knowledgeability"[All Fields] OR "knowledgeable"[All Fields] OR "knowledgeably"[All Fields] OR "knowledges"[All Fields]) OR ("attitude"[MeSH Terms] OR "attitude"[All Fields] OR "attitudes"[All Fields] OR "attitude s"[All Fields]) OR ("practicability"[All Fields] OR "practicable"[All Fields] OR "practical"[All Fields] OR "practicalities"[All Fields] OR "practicality"[All Fields] OR "practically"[All Fields] OR "practicals"[All Fields] OR "practice"[All Fields] OR "practice s"[All Fields] OR "practiced"[All Fields] OR "practices"[All Fields] OR "practicing"[All Fields]) OR ("awareness"[MeSH Terms] OR "awareness"[All Fields] OR "aware"[All Fields] OR "awarenesses"[All Fields]) OR "mobils*"[All Fields] OR "mobiliz*"[All Fields] OR "engage*"[All Fields] OR "empower*"[All Fields] OR "capacity building"[All Fields] OR ("education"[MeSH Subheading] OR "education"[All Fields] OR "training"[All Fields] OR "education"[MeSH Terms] OR "train"[All Fields] OR "train s"[All Fields] OR "trained"[All Fields] OR "training s"[All Fields] OR "trainings"[All Fields] OR "trains"[All Fields]) OR ("social behavior"[MeSH Terms] OR ("social"[All Fields] AND "behavior"[All Fields]) OR "social behavior"[All Fields] OR "sociality"[All Fields] OR "social"[All Fields] OR "socialisation"[All Fields] OR "socialization"[MeSH Terms] OR "socialization"[All Fields] OR "socialise"[All Fields] OR "socialised"[All Fields] OR "socialising"[All Fields] OR "socialities"[All Fields] OR "socializations"[All Fields] OR "socialize"[All Fields] OR "socialized"[All Fields] OR "socializers"[All Fields] OR "socializes"[All Fields] OR "socializing"[All Fields] OR "socially"[All Fields] OR "socials"[All Fields]) OR "community based strategy"[All Fields] OR "community based intervention"[All Fields] OR "community intervention"[All Fields]

Outcome:

"COVID 19"[All Fields] OR ("sars cov 2"[MeSH Terms] OR "sars cov 2"[All Fields] OR "covid"[All Fields] OR "COVID 19"[MeSH Terms] OR "COVID 19"[All Fields]) OR "Corona virus"[All Fields] OR ("tuberculosi"[All Fields] OR "tuberculosis"[MeSH Terms] OR "tuberculosis"[All Fields] OR "tuberculoses"[All Fields] OR "tuberculosis s"[All Fields]) OR "TB"[All Fields] OR ("hepatitis"[MeSH Terms] OR "hepatitis"[All Fields] OR "hepatitides"[All Fields] OR "hepatitis a"[MeSH Terms] OR "hepatitis a"[All Fields]) OR ("diarrhea"[MeSH Terms] OR "diarrhea"[All Fields] OR "diarrheas"[All Fields] OR "diarrhoea"[All Fields] OR "diarrhoeas"[All Fields]) OR ("typhoid fever"[MeSH Terms] OR ("typhoid"[All Fields] AND "fever"[All Fields]) OR "typhoid fever"[All Fields] OR "typhoid"[All Fields] OR "typhoidal"[All Fields]) OR ("dengue"[MeSH Terms] OR "dengue"[All Fields] OR "dengue s"[All Fields]) OR ("influenza s"[All Fields] OR "influenza, human"[MeSH Terms] OR ("influenza"[All Fields] AND "human"[All Fields]) OR "human influenza"[All Fields] OR "influenza"[All Fields] OR "influenzae"[All Fields] OR "influenzas"[All Fields]) OR ("pneumonia"[MeSH Terms] OR "pneumonia"[All Fields] OR "pneumoniae"[All Fields] OR "pneumonias"[All Fields] OR "pneumoniae s"[All Fields])

Final binding:=8,492

((((((("emerging country" [all fields] OR "emerging countries" [all fields] OR "emerging nation"[all fields] OR "emerging nations" [all fields] OR "emerging population"[all fields] OR "emerging populations"[all fields] OR "developing country"[tiab] OR "developing countries"[tiab] OR "developing nation"[tiab] OR "developing nations"[tiab] OR "developing population"[tiab] OR "developing populations"[tiab] OR "developing world"[tiab] OR "less developed country"[tiab] OR "less developed countries"[tiab] OR "less developed nation"[tiab] OR "less developed nations"[tiab] OR "less developed population"[tiab] OR "less developed populations"[tiab] OR "less developed world"[tiab] OR "lesser developed country"[tiab] OR "lesser developed countries"[tiab] OR "lesser developed nation"[tiab] OR "lesser developed nations"[tiab] OR "lesser developed population"[tiab] OR "lesser developed populations"[tiab] OR "lesser developed world"[tiab] OR "under developed country"[tiab] OR "under developed countries"[tiab] OR "under developed nation"[tiab] OR "under developed nations"[tiab] OR "under developed population"[tiab] OR "under developed populations"[tiab] OR "under developed world"[tiab] OR "underdeveloped country"[tiab] OR "underdeveloped countries"[tiab] OR "underdeveloped nation"[tiab] OR "underdeveloped nations"[tiab] OR "underdeveloped population"[tiab] OR "underdeveloped populations"[tiab] OR "underdeveloped world"[tiab] OR "middle income country"[tiab] OR "middle income countries"[tiab] OR "middle income nation"[tiab] OR "middle income nations"[tiab] OR "middle income population"[tiab] OR "middle income populations"[tiab] OR "low income country"[tiab] OR "low income countries"[tiab] OR "low income nation"[tiab] OR "low income nations"[tiab] OR "low income population"[tiab] OR "low income populations"[tiab] OR "lower income country"[tiab] OR "lower income countries"[tiab] OR "lower income nation"[tiab] OR "lower income nations"[tiab] OR "lower income population"[tiab] OR "lower income populations"[tiab] OR "underserved country"[tiab] OR "underserved countries"[tiab] OR "underserved nation"[tiab] OR "underserved nations"[tiab] OR "underserved population"[tiab] OR "underserved populations"[tiab] OR"underserved world"[tiab] OR "under served country"[tiab] OR "under served countries"[tiab] OR "under served nation"[tiab] OR "under served nations"[tiab] OR "under served population"[tiab] OR "under served populations"[tiab] OR "under served world"[tiab] OR "deprived country"[tiab] OR "deprived countries"[tiab] OR "deprived nation"[tiab] OR "deprived nations"[tiab] OR "deprived population"[tiab] OR "deprived populations"[tiab] OR "deprived world"[tiab] OR "poor country"[tiab] OR "poor countries"[tiab] OR "poor nation"[tiab] OR "poor nations"[tiab] OR "poor population"[tiab] OR "poor populations"[tiab] OR "poor world"[tiab] OR "poorer country"[tiab] OR "poorer countries"[tiab] OR "poorer nation"[tiab] OR "poorer nations"[tiab] OR "poorer population"[tiab] OR "poorer populations"[tiab] OR "poorer world"[tiab] OR "developing economy"[tiab] OR "developing economies"[tiab] OR "less developed economy"[tiab] OR "less developed economies"[tiab] OR "lesser developed economy"[tiab] OR "lesser developed economies"[tiab] OR "under developed economy"[tiab] OR "under developed economies"[tiab] OR "underdeveloped economy"[tiab] OR "underdeveloped economies"[tiab] OR "middle income economy"[tiab] OR "middle income economies"[tiab] OR "low income economy"[tiab] OR "low income economies"[tiab] OR "lower income economy"[tiab] OR "lower income economies"[tiab] OR "low gdp"[tiab] OR "low gnp"[tiab] OR "low gross domestic"[tiab] OR "low gross national"[tiab] OR "lower gdp"[tiab] OR "lower gnp"[tiab] OR "lower gross domestic"[tiab] OR "lower gross national"[tiab] OR lmic[tiab] OR lmics[tiab] OR "third world"[tiab] OR "lami country"[tiab] OR "lami countries"[tiab] OR "transitional country"[tiab] OR "transitional countries"[tiab] OR Africa[tiab] OR Asia[tiab] OR Caribbean[tiab] OR West Indies[tiab] OR South America[tiab] OR Latin America[tiab] OR Central America[tiab] OR "Atlantic Islands"[tiab] OR "Commonwealth of Independent States"[tiab] OR "Pacific Islands"[tiab] OR "Indian Ocean Islands"[tiab] OR Afghanistan[tiab] OR Albania[tiab] OR Algeria[tiab] OR Angola[tiab] OR Antigua[tiab] OR Barbuda[tiab] OR Argentina[tiab] OR OR Aruba[tiab] OR Azerbaijan[tiab] OR Bangladesh[tiab] OR Barbados[tiab] OR Benin[tiab] OR Byelarus[tiab] OR Byelorussian[tiab] OR Belarus[tiab] OR Belorussian[tiab] OR Belorussia[tiab] OR Belize[tiab] OR Bhutan[tiab] OR Bolivia[tiab] OR Bosnia[tiab] OR Herzegovina[tiab] OR Hercegovina[tiab] OR Botswana[tiab] OR Brasil[tiab] OR Brazil[tiab] OR Bulgaria[tiab] OR Burkina Faso[tiab] OR Burkina Fasso[tiab] OR Upper Volta[tiab] OR Burundi[tiab] OR Urundi[tiab] OR Cambodia[tiab] OR Khmer Republic[tiab] OR Kampuchea[tiab] OR Cameroon[tiab] OR Cameroons[tiab] OR Cameron[tiab] OR Cape Verde[tiab] OR Central African Republic[tiab] OR Chad[tiab] OR China[tiab] OR Colombia[tiab] OR Comoros[tiab] OR Comoro Islands[tiab] OR Comores[tiab] OR Congo[tiab] OR Zaire[tiab] OR Costa Rica[tiab] OR Cote d'Ivoire[tiab] OR Ivory Coast[tiab] OR Cuba[tiab] OR Cyprus[tiab] OR Czechoslovakia[tiab] OR "Czech Republic" [tiab] OR Slovakia[tiab] OR Slovak Republic[tiab] OR Dominica[tiab] OR Dominican Republic[tiab] OR East Timor[tiab] OR East Timur[tiab] OR Timor Leste[tiab] OR Ecuador[tiab] OR \ OR El Salvador[tiab] OR Eritrea[tiab] OR Estonia[tiab] OR Ethiopia[tiab] OR Fiji[tiab] OR Gabon[tiab] OR Gabonese Republic[tiab] OR Gambia[tiab] OR Gaza[tiab] OR Georgia Republic[tiab] OR Georgian Republic[tiab] OR Ghana[tiab] OR Gold Coast[tiab] OR Greece[tiab] OR Grenada[tiab] OR Guatemala[tiab] OR Guinea[tiab] OR Guam[tiab] OR Guiana[tiab] OR Guyana[tiab] OR Haiti[tiab] OR Honduras[tiab] OR Hungary[tiab] OR India[tiab] OR Maldives[tiab] OR Indonesia[tiab] OR Jamaica[tiab] OR Kazakhstan[tiab] OR Kazakh[tiab] OR Kenya[tiab] OR Kiribati[tiab] OR Korea[tiab] OR Kosovo[tiab] OR Kyrgyzstan[tiab] OR Kirghizia[tiab] OR Kyrgyz Republic[tiab] OR Kirghiz[tiab] OR Kirgizstan[tiab] OR "Lao PDR"[tiab] OR Laos[tiab] OR Latvia[tiab] OR Lebanon[tiab] OR Lesotho[tiab] OR Basutoland[tiab] OR Liberia[tiab] OR Lithuania[tiab]OR Macedonia[tiab] OR Madagascar[tiab] OR Malagasy Republic[tiab] OR Malaysia[tiab] OR Malaya[tiab] OR Malay[tiab] OR Sabah[tiab] OR Sarawak[tiab] OR Malawi[tiab] OR Nyasaland[tiab] OR Mali[tiab] OR Marshall Islands[tiab] OR Mauritania[tiab] OR Mauritius[tiab] OR Agalega Islands[tiab] OR "Melanesia"[tiab] OR Mexico[tiab] OR Micronesia[tiab] OR Middle East[tiab] OR Moldova[tiab] OR Moldovia[tiab] OR Moldovian[tiab] OR Mongolia[tiab] OR Montenegro[tiab] OR Mozambique[tiab] OR Myanmar[tiab] OR Myanma[tiab] OR Burma[tiab] OR Namibia[tiab] OR Nepal[tiab] OR Netherlands Antilles[tiab] OR New Caledonia[tiab] OR Nicaragua[tiab] OR Niger[tiab] OR Nigeria[tiab] OR Northern Mariana Islands[tiab] OR OR Pakistan[tiab] OR Palau[tiab] OR Palestine[tiab] OR Panama[tiab] OR Paraguay[tiab] OR Peru[tiab] OR Philippines[tiab] OR Philipines[tiab] OR Phillipines[tiab] OR Phillippines[tiab] OR Poland[tiab] OR Portugal[tiab] OR Puerto Rico[tiab] OR Russia[tiab] OR Russian[tiab] OR Rwanda[tiab] OR Ruanda[tiab] OR Saint Kitts[tiab] OR St Kitts[tiab] OR Nevis[tiab] OR Saint Lucia[tiab] OR St Lucia[tiab] OR Saint Vincent[tiab] OR St Vincent[tiab] OR Grenadines[tiab] OR Samoa[tiab] OR Samoan Islands[tiab] OR Navigator Island[tiab] OR Navigator Islands[tiab] OR Sao Tome[tiab] OR Senegal[tiab] OR Serbia[tiab] OR Montenegro[tiab] OR Seychelles[tiab] OR Sierra Leone[tiab] OR Slovenia[tiab] OR Sri Lanka[tiab] OR Ceylon[tiab] OR Solomon Islands[tiab] OR Somalia[tiab] OR Sudan[tiab] OR Suriname[tiab] OR Surinam[tiab] OR Swaziland[tiab] OR Tajikistan[tiab] OR Tadzhikistan[tiab] OR Tadjikistan[tiab] OR Tadzhik[tiab] OR Tanzania[tiab] OR Thailand[tiab] OR Togo[tiab] OR Togolese Republic[tiab] OR Tonga[tiab] OR Trinidad[tiab] OR Tobago[tiab] OR Turkey[tiab] OR Turkmenistan[tiab] OR Turkmen[tiab] OR Tuvalu[tiab] OR Uganda[tiab] OR Ukraine[tiab] OR Uruguay[tiab] OR USSR[tiab] OR Soviet Union[tiab] OR Union of Soviet Socialist Republics[tiab] OR Uzbekistan[tiab] OR Uzbek OR Vanuatu[tiab] OR New Hebrides[tiab] OR Venezuela[tiab] OR Vietnam[tiab] OR Viet Nam[tiab] OR Zambia[tiab] OR Zimbabwe[tiab] OR Rhodesia[tiab] OR Developing Countries[Mesh] OR Africa[Mesh:NoExp] OR Africa South of the Sahara[Mesh:NoExp] OR Africa, Central[Mesh:NoExp] OR Africa, Eastern[Mesh:NoExp] OR Africa, Southern[Mesh:NoExp] OR Africa, Western[Mesh:NoExp] OR Asia[Mesh:NoExp] OR Asia, Central[Mesh:NoExp] OR Asia, Southeastern[Mesh:NoExp] OR Asia, Western[Mesh:NoExp] OR Caribbean Region[Mesh:NoExp] OR West Indies[Mesh:NoExp] OR South America[Mesh:NoExp] OR Latin America[Mesh:NoExp] OR Central America[Mesh:NoExp] OR "Atlantic Islands"[Mesh:NoExp] OR "Commonwealth of Independent States"[Mesh:NoExp] OR "Pacific Islands"[Mesh:NoExp] OR "Indian Ocean Islands"[Mesh:NoExp] OR Afghanistan[Mesh] OR Albania[Mesh] OR Algeria[Mesh] OR American Samoa[Mesh] OR Angola[Mesh] OR "Antigua and Barbuda"[Mesh] OR Argentina[Mesh] OR Azerbaijan[Mesh] OR Bahrain[Mesh] OR "Baltic States"[Mesh] OR Bangladesh[Mesh] OR Barbados[Mesh] OR Benin[Mesh] OR "Republic of Belarus"[Mesh] OR Belize[Mesh] OR Bhutan[Mesh] OR Bolivia[Mesh] OR Bosnia-Herzegovina[Mesh] OR Botswana[Mesh] OR Brazil[Mesh] OR Bulgaria[Mesh] OR Burkina Faso[Mesh] OR Burundi[Mesh] OR Cambodia[Mesh] OR Cameroon[Mesh] OR Cape Verde[Mesh] OR Central African Republic[Mesh] OR Chad[Mesh] OR Chile[Mesh] OR China[Mesh] OR Colombia[Mesh] OR Comoros[Mesh] OR Congo[Mesh] OR Costa Rica[Mesh] OR Cote d'Ivoire[Mesh] OR Croatia[Mesh] OR Cuba[Mesh] OR Cyprus[Mesh] OR Czechoslovakia[Mesh] OR Czech Republic[Mesh] OR Slovakia[Mesh] OR "Democratic Republic of the Congo"[Mesh] OR "Democratic People's Republic of Korea"[Mesh] OR Dominica[Mesh] OR Dominican Republic[Mesh] OR East Timor[Mesh] OR Ecuador[Mesh] OR El Salvador[Mesh] OR Eritrea[Mesh] OR Estonia[Mesh] OR Ethiopia[Mesh] OR "Equatorial Guinea"[Mesh] OR Fiji[Mesh] OR "French Guiana"[Mesh] OR Gabon[Mesh] OR Gambia[Mesh] OR "Georgia (Republic)"[Mesh] OR Ghana[Mesh] OR Greece[Mesh] OR Grenada[Mesh] OR Guatemala[Mesh] OR Guinea[Mesh] OR Guinea-Bissau[Mesh] OR Guam[Mesh] OR Guyana[Mesh] OR Haiti[Mesh] OR Honduras[Mesh] OR Hungary[Mesh] OR "Independent State of Samoa"[Mesh] OR India[Mesh] OR Indonesia[Mesh] OR Jamaica[Mesh] OR Kazakhstan[Mesh] OR Kenya[Mesh] OR Korea[Mesh] OR Kyrgyzstan[Mesh] OR Laos[Mesh] OR Latvia[Mesh] OR Lebanon[Mesh] OR Lesotho[Mesh] OR Liberia[Mesh] OR Lithuania[Mesh] OR "Macedonia (Republic)"[Mesh] OR Madagascar[Mesh] OR Malawi[Mesh] OR Malaysia[Mesh] OR Mali[Mesh] OR Mauritania[Mesh] OR Mauritius[Mesh] OR "Melanesia"[Mesh] OR Mexico[Mesh] OR Micronesia[Mesh] OR Middle East[Mesh:NoExp] ORMoldova[Mesh] OR Mongolia[Mesh] OR Montenegro[Mesh] OR Mozambique[Mesh] OR Myanmar[Mesh] OR Namibia[Mesh] OR Nepal[Mesh] OR Netherlands Antilles[Mesh] OR New Caledonia[Mesh] OR Nicaragua[Mesh] OR Niger[Mesh] OR Nigeria[Mesh] OR Pakistan[Mesh] OR Palau[Mesh] OR Panama[Mesh] OR Papua New Guinea[Mesh] OR Paraguay[Mesh] OR Peru[Mesh] OR Philippines[Mesh] OR Poland[Mesh] OR Portugal[Mesh] OR Puerto Rico[Mesh] OR "Republic of Korea"[Mesh] OR Romania[Mesh] OR Russia[Mesh] OR "Russia (Pre-1917)"[Mesh] OR Rwanda[Mesh] OR "Saint Kitts and Nevis"[Mesh] OR Saint Lucia[Mesh] OR "Saint Vincent and the Grenadines"[Mesh] OR Samoa[Mesh] OR Saudi Arabia[Mesh] OR Senegal[Mesh] OR Serbia[Mesh] OR Montenegro[Mesh] OR Seychelles[Mesh] OR Sierra Leone[Mesh] OR Slovenia[Mesh] OR Sri Lanka[Mesh] OR Somalia[Mesh] OR South Africa[Mesh] OR Sudan[Mesh] OR Suriname[Mesh] OR Swaziland[Mesh] OR Syria[Mesh] OR Tajikistan[Mesh] OR Tanzania[Mesh] OR Thailand[Mesh] OR Togo[Mesh] OR Tonga[Mesh] OR "Trinidad and Tobago"[Mesh] OR Tunisia[Mesh] OR Turkey[Mesh] OR Turkmenistan[Mesh] OR Uganda[Mesh] OR Ukraine[Mesh] OR Uruguay[Mesh] OR USSR[Mesh] OR Uzbekistan[Mesh] OR Vanuatu[Mesh] OR Venezuela[Mesh] OR Vietnam[Mesh] OR Yemen[Mesh] OR Yugoslavia[Mesh] OR Zambia[Mesh] OR Zimbabwe[Mesh] OR "Southern African Development Community" [all fields] OR "East African Community"[all fields] OR "West African Health Organisation"[all fields] OR "Sub Saharan Africa "[all fields] OR "SubSaharan Africa "[all fields])) OR (urban)) OR (((((slum) OR ("informal settlement")) OR ("informal settlements")) OR (poor)) OR (disadvantage*))) OR ("poverty areas"[MeSH Terms] OR ("poverty"[All Fields] AND "areas"[All Fields]) OR "poverty areas"[All Fields] OR "slum"[All Fields] OR "informal settlement"[All Fields] OR "informal settlements"[All Fields] OR ("poverty"[MeSH Terms] OR "poverty"[All Fields] OR "poor"[All Fields]) OR "disadvantage*"[All Fields])) AND ("prevent*"[All Fields] OR ("controling"[All Fields] OR "controllability"[All Fields] OR "controllable"[All Fields] OR "controllably"[All Fields] OR "controller"[All Fields] OR "controller s"[All Fields] OR "controllers"[All Fields] OR "controlling"[All Fields] OR "controls"[All Fields] OR "prevention and control"[MeSH Subheading] OR ("prevention"[All Fields] AND "control"[All Fields]) OR "prevention and control"[All Fields] OR "control"[All Fields] OR "control groups"[MeSH Terms] OR ("control"[All Fields] AND "groups"[All Fields]) OR "control groups"[All Fields]) OR "communit*"[All Fields] OR ("knowledge"[MeSH Terms] OR "knowledge"[All Fields] OR "knowledge s"[All Fields] OR "knowledgeability"[All Fields] OR "knowledgeable"[All Fields] OR "knowledgeably"[All Fields] OR "knowledges"[All Fields]) OR ("attitude"[MeSH Terms] OR "attitude"[All Fields] OR "attitudes"[All Fields] OR "attitude s"[All Fields]) OR ("practicability"[All Fields] OR "practicable"[All Fields] OR "practical"[All Fields] OR "practicalities"[All Fields] OR "practicality"[All Fields] OR "practically"[All Fields] OR "practicals"[All Fields] OR "practice"[All Fields] OR "practice s"[All Fields] OR "practiced"[All Fields] OR "practices"[All Fields] OR "practicing"[All Fields]) OR ("awareness"[MeSH Terms] OR "awareness"[All Fields] OR "aware"[All Fields] OR "awarenesses"[All Fields]) OR "mobils*"[All Fields] OR "mobiliz*"[All Fields] OR "engage*"[All Fields] OR "empower*"[All Fields] OR "capacity building"[All Fields] OR ("education"[MeSH Subheading] OR "education"[All Fields] OR "training"[All Fields] OR "education"[MeSH Terms] OR "train"[All Fields] OR "train s"[All Fields] OR "trained"[All Fields] OR "training s"[All Fields] OR "trainings"[All Fields] OR "trains"[All Fields]) OR ("social behavior"[MeSH Terms] OR ("social"[All Fields] AND "behavior"[All Fields]) OR "social behavior"[All Fields] OR "sociality"[All Fields] OR "social"[All Fields] OR "socialisation"[All Fields] OR "socialization"[MeSH Terms] OR "socialization"[All Fields] OR "socialise"[All Fields] OR "socialised"[All Fields] OR "socialising"[All Fields] OR "socialities"[All Fields] OR "socializations"[All Fields] OR "socialize"[All Fields] OR "socialized"[All Fields] OR "socializers"[All Fields] OR "socializes"[All Fields] OR "socializing"[All Fields] OR "socially"[All Fields] OR "socials"[All Fields]) OR "community based strategy"[All Fields] OR "community based intervention"[All Fields] OR "community intervention"[All Fields])) AND ((((((((((("COVID 19") OR (COVID)) OR ("Corona virus")) OR (tuberculosis)) OR (TB)) OR (Hepatitis)) OR (diarrhoea)) OR (Typhoid)) OR (dengue)) OR (influenza)) OR (Pneumonia))) NOT (((survey) OR ("clinical trial")) OR ("drug treatment"))

Filter used:

English, Human, Publication year (1^st^ January 2011 – 30^th^ September, 2021), age: (adult 19+), journal type (RCT, evaluation study, journal article)

(((((((("emerging country" [all fields] OR "emerging countries" [all fields] OR "emerging nation"[all fields] OR "emerging nations" [all fields] OR "emerging population"[all fields] OR "emerging populations"[all fields] OR "developing country"[tiab] OR "developing countries"[tiab] OR "developing nation"[tiab] OR "developing nations"[tiab] OR "developing population"[tiab] OR "developing populations"[tiab] OR "developing world"[tiab] OR "less developed country"[tiab] OR "less developed countries"[tiab] OR "less developed nation"[tiab] OR "less developed nations"[tiab] OR "less developed population"[tiab] OR "less developed populations"[tiab] OR "less developed world"[tiab] OR "lesser developed country"[tiab] OR "lesser developed countries"[tiab] OR "lesser developed nation"[tiab] OR "lesser developed nations"[tiab] OR "lesser developed population"[tiab] OR "lesser developed populations"[tiab] OR "lesser developed world"[tiab] OR "under developed country"[tiab] OR "under developed countries"[tiab] OR "under developed nation"[tiab] OR "under developed nations"[tiab] OR "under developed population"[tiab] OR "under developed populations"[tiab] OR "under developed world"[tiab] OR "underdeveloped country"[tiab] OR "underdeveloped countries"[tiab] OR "underdeveloped nation"[tiab] OR "underdeveloped nations"[tiab] OR "underdeveloped population"[tiab] OR "underdeveloped populations"[tiab] OR "underdeveloped world"[tiab] OR "middle income country"[tiab] OR "middle income countries"[tiab] OR "middle income nation"[tiab] OR "middle income nations"[tiab] OR "middle income population"[tiab] OR "middle income populations"[tiab] OR "low income country"[tiab] OR "low income countries"[tiab] OR "low income nation"[tiab] OR "low income nations"[tiab] OR "low income population"[tiab] OR "low income populations"[tiab] OR "lower income country"[tiab] OR "lower income countries"[tiab] OR "lower income nation"[tiab] OR "lower income nations"[tiab] OR "lower income population"[tiab] OR "lower income populations"[tiab] OR "underserved country"[tiab] OR "underserved countries"[tiab] OR "underserved nation"[tiab] OR "underserved nations"[tiab] OR "underserved population"[tiab] OR "underserved populations"[tiab] OR"underserved world"[tiab] OR "under served country"[tiab] OR "under served countries"[tiab] OR "under served nation"[tiab] OR "under served nations"[tiab] OR "under served population"[tiab] OR "under served populations"[tiab] OR "under served world"[tiab] OR "deprived country"[tiab] OR "deprived countries"[tiab] OR "deprived nation"[tiab] OR "deprived nations"[tiab] OR "deprived population"[tiab] OR "deprived populations"[tiab] OR "deprived world"[tiab] OR "poor country"[tiab] OR "poor countries"[tiab] OR "poor nation"[tiab] OR "poor nations"[tiab] OR "poor population"[tiab] OR "poor populations"[tiab] OR "poor world"[tiab] OR "poorer country"[tiab] OR "poorer countries"[tiab] OR "poorer nation"[tiab] OR "poorer nations"[tiab] OR "poorer population"[tiab] OR "poorer populations"[tiab] OR "poorer world"[tiab] OR "developing economy"[tiab] OR "developing economies"[tiab] OR "less developed economy"[tiab] OR "less developed economies"[tiab] OR "lesser developed economy"[tiab] OR "lesser developed economies"[tiab] OR "under developed economy"[tiab] OR "under developed economies"[tiab] OR "underdeveloped economy"[tiab] OR "underdeveloped economies"[tiab] OR "middle income economy"[tiab] OR "middle income economies"[tiab] OR "low income economy"[tiab] OR "low income economies"[tiab] OR "lower income economy"[tiab] OR "lower income economies"[tiab] OR "low gdp"[tiab] OR "low gnp"[tiab] OR "low gross domestic"[tiab] OR "low gross national"[tiab] OR "lower gdp"[tiab] OR "lower gnp"[tiab] OR "lower gross domestic"[tiab] OR "lower gross national"[tiab] OR lmic[tiab] OR lmics[tiab] OR "third world"[tiab] OR "lami country"[tiab] OR "lami countries"[tiab] OR "transitional country"[tiab] OR "transitional countries"[tiab] OR Africa[tiab] OR Asia[tiab] OR Caribbean[tiab] OR West Indies[tiab] OR South America[tiab] OR Latin America[tiab] OR Central America[tiab] OR "Atlantic Islands"[tiab] OR "Commonwealth of Independent States"[tiab] OR "Pacific Islands"[tiab] OR "Indian Ocean Islands"[tiab] OR Afghanistan[tiab] OR Albania[tiab] OR Algeria[tiab] OR Angola[tiab] OR Antigua[tiab] OR Barbuda[tiab] OR Argentina[tiab] OR OR Aruba[tiab] OR Azerbaijan[tiab] OR Bangladesh[tiab] OR Barbados[tiab] OR Benin[tiab] OR Byelarus[tiab] OR Byelorussian[tiab] OR Belarus[tiab] OR Belorussian[tiab] OR Belorussia[tiab] OR Belize[tiab] OR Bhutan[tiab] OR Bolivia[tiab] OR Bosnia[tiab] OR Herzegovina[tiab] OR Hercegovina[tiab] OR Botswana[tiab] OR Brasil[tiab] OR Brazil[tiab] OR Bulgaria[tiab] OR Burkina Faso[tiab] OR Burkina Fasso[tiab] OR Upper Volta[tiab] OR Burundi[tiab] OR Urundi[tiab] OR Cambodia[tiab] OR Khmer Republic[tiab] OR Kampuchea[tiab] OR Cameroon[tiab] OR Cameroons[tiab] OR Cameron[tiab] OR Cape Verde[tiab] OR Central African Republic[tiab] OR Chad[tiab] OR China[tiab] OR Colombia[tiab] OR Comoros[tiab] OR Comoro Islands[tiab] OR Comores[tiab] OR Congo[tiab] OR Zaire[tiab] OR Costa Rica[tiab] OR Cote d'Ivoire[tiab] OR Ivory Coast[tiab] OR Cuba[tiab] OR Cyprus[tiab] OR Czechoslovakia[tiab] OR "Czech Republic" [tiab] OR Slovakia[tiab] OR Slovak Republic[tiab] OR Dominica[tiab] OR Dominican Republic[tiab] OR East Timor[tiab] OR East Timur[tiab] OR Timor Leste[tiab] OR Ecuador[tiab] OR \ OR El Salvador[tiab] OR Eritrea[tiab] OR Estonia[tiab] OR Ethiopia[tiab] OR Fiji[tiab] OR Gabon[tiab] OR Gabonese Republic[tiab] OR Gambia[tiab] OR Gaza[tiab] OR Georgia Republic[tiab] OR Georgian Republic[tiab] OR Ghana[tiab] OR Gold Coast[tiab] OR Greece[tiab] OR Grenada[tiab] OR Guatemala[tiab] OR Guinea[tiab] OR Guam[tiab] OR Guiana[tiab] OR Guyana[tiab] OR Haiti[tiab] OR Honduras[tiab] OR Hungary[tiab] OR India[tiab] OR Maldives[tiab] OR Indonesia[tiab] OR Jamaica[tiab] OR Kazakhstan[tiab] OR Kazakh[tiab] OR Kenya[tiab] OR Kiribati[tiab] OR Korea[tiab] OR Kosovo[tiab] OR Kyrgyzstan[tiab] OR Kirghizia[tiab] OR Kyrgyz Republic[tiab] OR Kirghiz[tiab] OR Kirgizstan[tiab] OR "Lao PDR"[tiab] OR Laos[tiab] OR Latvia[tiab] OR Lebanon[tiab] OR Lesotho[tiab] OR Basutoland[tiab] OR Liberia[tiab] OR Lithuania[tiab]OR Macedonia[tiab] OR Madagascar[tiab] OR Malagasy Republic[tiab] OR Malaysia[tiab] OR Malaya[tiab] OR Malay[tiab] OR Sabah[tiab] OR Sarawak[tiab] OR Malawi[tiab] OR Nyasaland[tiab] OR Mali[tiab] OR Marshall Islands[tiab] OR Mauritania[tiab] OR Mauritius[tiab] OR Agalega Islands[tiab] OR "Melanesia"[tiab] OR Mexico[tiab] OR Micronesia[tiab] OR Middle East[tiab] OR Moldova[tiab] OR Moldovia[tiab] OR Moldovian[tiab] OR Mongolia[tiab] OR Montenegro[tiab] OR Mozambique[tiab] OR Myanmar[tiab] OR Myanma[tiab] OR Burma[tiab] OR Namibia[tiab] OR Nepal[tiab] OR Netherlands Antilles[tiab] OR New Caledonia[tiab] OR Nicaragua[tiab] OR Niger[tiab] OR Nigeria[tiab] OR Northern Mariana Islands[tiab] OR OR Pakistan[tiab] OR Palau[tiab] OR Palestine[tiab] OR Panama[tiab] OR Paraguay[tiab] OR Peru[tiab] OR Philippines[tiab] OR Philipines[tiab] OR Phillipines[tiab] OR Phillippines[tiab] OR Poland[tiab] OR Portugal[tiab] OR Puerto Rico[tiab] OR Russia[tiab] OR Russian[tiab] OR Rwanda[tiab] OR Ruanda[tiab] OR Saint Kitts[tiab] OR St Kitts[tiab] OR Nevis[tiab] OR Saint Lucia[tiab] OR St Lucia[tiab] OR Saint Vincent[tiab] OR St Vincent[tiab] OR Grenadines[tiab] OR Samoa[tiab] OR Samoan Islands[tiab] OR Navigator Island[tiab] OR Navigator Islands[tiab] OR Sao Tome[tiab] OR Senegal[tiab] OR Serbia[tiab] OR Montenegro[tiab] OR Seychelles[tiab] OR Sierra Leone[tiab] OR Slovenia[tiab] OR Sri Lanka[tiab] OR Ceylon[tiab] OR Solomon Islands[tiab] OR Somalia[tiab] OR Sudan[tiab] OR Suriname[tiab] OR Surinam[tiab] OR Swaziland[tiab] OR Tajikistan[tiab] OR Tadzhikistan[tiab] OR Tadjikistan[tiab] OR Tadzhik[tiab] OR Tanzania[tiab] OR Thailand[tiab] OR Togo[tiab] OR Togolese Republic[tiab] OR Tonga[tiab] OR Trinidad[tiab] OR Tobago[tiab] OR Turkey[tiab] OR Turkmenistan[tiab] OR Turkmen[tiab] OR Tuvalu[tiab] OR Uganda[tiab] OR Ukraine[tiab] OR Uruguay[tiab] OR USSR[tiab] OR Soviet Union[tiab] OR Union of Soviet Socialist Republics[tiab] OR Uzbekistan[tiab] OR Uzbek OR Vanuatu[tiab] OR New Hebrides[tiab] OR Venezuela[tiab] OR Vietnam[tiab] OR Viet Nam[tiab] OR Zambia[tiab] OR Zimbabwe[tiab] OR Rhodesia[tiab] OR Developing Countries[Mesh] OR Africa[Mesh:NoExp] OR Africa South of the Sahara[Mesh:NoExp] OR Africa, Central[Mesh:NoExp] OR Africa, Eastern[Mesh:NoExp] OR Africa, Southern[Mesh:NoExp] OR Africa, Western[Mesh:NoExp] OR Asia[Mesh:NoExp] OR Asia, Central[Mesh:NoExp] OR Asia, Southeastern[Mesh:NoExp] OR Asia, Western[Mesh:NoExp] OR Caribbean Region[Mesh:NoExp] OR West Indies[Mesh:NoExp] OR South America[Mesh:NoExp] OR Latin America[Mesh:NoExp] OR Central America[Mesh:NoExp] OR "Atlantic Islands"[Mesh:NoExp] OR "Commonwealth of Independent States"[Mesh:NoExp] OR "Pacific Islands"[Mesh:NoExp] OR "Indian Ocean Islands"[Mesh:NoExp] OR Afghanistan[Mesh] OR Albania[Mesh] OR Algeria[Mesh] OR American Samoa[Mesh] OR Angola[Mesh] OR "Antigua and Barbuda"[Mesh] OR Argentina[Mesh] OR Azerbaijan[Mesh] OR Bahrain[Mesh] OR "Baltic States"[Mesh] OR Bangladesh[Mesh] OR Barbados[Mesh] OR Benin[Mesh] OR "Republic of Belarus"[Mesh] OR Belize[Mesh] OR Bhutan[Mesh] OR Bolivia[Mesh] OR Bosnia-Herzegovina[Mesh] OR Botswana[Mesh] OR Brazil[Mesh] OR Bulgaria[Mesh] OR Burkina Faso[Mesh] OR Burundi[Mesh] OR Cambodia[Mesh] OR Cameroon[Mesh] OR Cape Verde[Mesh] OR Central African Republic[Mesh] OR Chad[Mesh] OR Chile[Mesh] OR China[Mesh] OR Colombia[Mesh] OR Comoros[Mesh] OR Congo[Mesh] OR Costa Rica[Mesh] OR Cote d'Ivoire[Mesh] OR Croatia[Mesh] OR Cuba[Mesh] OR Cyprus[Mesh] OR Czechoslovakia[Mesh] OR Czech Republic[Mesh] OR Slovakia[Mesh] OR "Democratic Republic of the Congo"[Mesh] OR "Democratic People's Republic of Korea"[Mesh] OR Dominica[Mesh] OR Dominican Republic[Mesh] OR East Timor[Mesh] OR Ecuador[Mesh] OR El Salvador[Mesh] OR Eritrea[Mesh] OR Estonia[Mesh] OR Ethiopia[Mesh] OR "Equatorial Guinea"[Mesh] OR Fiji[Mesh] OR "French Guiana"[Mesh] OR Gabon[Mesh] OR Gambia[Mesh] OR "Georgia (Republic)"[Mesh] OR Ghana[Mesh] OR Greece[Mesh] OR Grenada[Mesh] OR Guatemala[Mesh] OR Guinea[Mesh] OR Guinea-Bissau[Mesh] OR Guam[Mesh] OR Guyana[Mesh] OR Haiti[Mesh] OR Honduras[Mesh] OR Hungary[Mesh] OR "Independent State of Samoa"[Mesh] OR India[Mesh] OR Indonesia[Mesh] OR Jamaica[Mesh] OR Kazakhstan[Mesh] OR Kenya[Mesh] OR Korea[Mesh] OR Kyrgyzstan[Mesh] OR Laos[Mesh] OR Latvia[Mesh] OR Lebanon[Mesh] OR Lesotho[Mesh] OR Liberia[Mesh] OR Lithuania[Mesh] OR "Macedonia (Republic)"[Mesh] OR Madagascar[Mesh] OR Malawi[Mesh] OR Malaysia[Mesh] OR Mali[Mesh] OR Mauritania[Mesh] OR Mauritius[Mesh] OR "Melanesia"[Mesh] OR Mexico[Mesh] OR Micronesia[Mesh] OR Middle East[Mesh:NoExp] ORMoldova[Mesh] OR Mongolia[Mesh] OR Montenegro[Mesh] OR Mozambique[Mesh] OR Myanmar[Mesh] OR Namibia[Mesh] OR Nepal[Mesh] OR Netherlands Antilles[Mesh] OR New Caledonia[Mesh] OR Nicaragua[Mesh] OR Niger[Mesh] OR Nigeria[Mesh] OR Pakistan[Mesh] OR Palau[Mesh] OR Panama[Mesh] OR Papua New Guinea[Mesh] OR Paraguay[Mesh] OR Peru[Mesh] OR Philippines[Mesh] OR Poland[Mesh] OR Portugal[Mesh] OR Puerto Rico[Mesh] OR "Republic of Korea"[Mesh] OR Romania[Mesh] OR Russia[Mesh] OR "Russia (Pre-1917)"[Mesh] OR Rwanda[Mesh] OR "Saint Kitts and Nevis"[Mesh] OR Saint Lucia[Mesh] OR "Saint Vincent and the Grenadines"[Mesh] OR Samoa[Mesh] OR Saudi Arabia[Mesh] OR Senegal[Mesh] OR Serbia[Mesh] OR Montenegro[Mesh] OR Seychelles[Mesh] OR Sierra Leone[Mesh] OR Slovenia[Mesh] OR Sri Lanka[Mesh] OR Somalia[Mesh] OR South Africa[Mesh] OR Sudan[Mesh] OR Suriname[Mesh] OR Swaziland[Mesh] OR Syria[Mesh] OR Tajikistan[Mesh] OR Tanzania[Mesh] OR Thailand[Mesh] OR Togo[Mesh] OR Tonga[Mesh] OR "Trinidad and Tobago"[Mesh] OR Tunisia[Mesh] OR Turkey[Mesh] OR Turkmenistan[Mesh] OR Uganda[Mesh] OR Ukraine[Mesh] OR Uruguay[Mesh] OR USSR[Mesh] OR Uzbekistan[Mesh] OR Vanuatu[Mesh] OR Venezuela[Mesh] OR Vietnam[Mesh] OR Yemen[Mesh] OR Yugoslavia[Mesh] OR Zambia[Mesh] OR Zimbabwe[Mesh] OR "Southern African Development Community" [all fields] OR "East African Community"[all fields] OR "West African Health Organisation"[all fields] OR "Sub Saharan Africa "[all fields] OR "SubSaharan Africa "[all fields])) OR (urban)) OR (((((slum) OR ("informal settlement")) OR ("informal settlements")) OR (poor)) OR (disadvantage*))) OR ("poverty areas"[MeSH Terms] OR ("poverty"[All Fields] AND "areas"[All Fields]) OR "poverty areas"[All Fields] OR "slum"[All Fields] OR "informal settlement"[All Fields] OR "informal settlements"[All Fields] OR ("poverty"[MeSH Terms] OR "poverty"[All Fields] OR "poor"[All Fields]) OR "disadvantage*"[All Fields])) AND ("prevent*"[All Fields] OR ("controling"[All Fields] OR "controllability"[All Fields] OR "controllable"[All Fields] OR "controllably"[All Fields] OR "controller"[All Fields] OR "controller s"[All Fields] OR "controllers"[All Fields] OR "controlling"[All Fields] OR "controls"[All Fields] OR "prevention and control"[MeSH Subheading] OR ("prevention"[All Fields] AND "control"[All Fields]) OR "prevention and control"[All Fields] OR "control"[All Fields] OR "control groups"[MeSH Terms] OR ("control"[All Fields] AND "groups"[All Fields]) OR "control groups"[All Fields]) OR "communit*"[All Fields] OR ("knowledge"[MeSH Terms] OR "knowledge"[All Fields] OR "knowledge s"[All Fields] OR "knowledgeability"[All Fields] OR "knowledgeable"[All Fields] OR "knowledgeably"[All Fields] OR "knowledges"[All Fields]) OR ("attitude"[MeSH Terms] OR "attitude"[All Fields] OR "attitudes"[All Fields] OR "attitude s"[All Fields]) OR ("practicability"[All Fields] OR "practicable"[All Fields] OR "practical"[All Fields] OR "practicalities"[All Fields] OR "practicality"[All Fields] OR "practically"[All Fields] OR "practicals"[All Fields] OR "practice"[All Fields] OR "practice s"[All Fields] OR "practiced"[All Fields] OR "practices"[All Fields] OR "practicing"[All Fields]) OR ("awareness"[MeSH Terms] OR "awareness"[All Fields] OR "aware"[All Fields] OR "awarenesses"[All Fields]) OR "mobils*"[All Fields] OR "mobiliz*"[All Fields] OR "engage*"[All Fields] OR "empower*"[All Fields] OR "capacity building"[All Fields] OR ("education"[MeSH Subheading] OR "education"[All Fields] OR "training"[All Fields] OR "education"[MeSH Terms] OR "train"[All Fields] OR "train s"[All Fields] OR "trained"[All Fields] OR "training s"[All Fields] OR "trainings"[All Fields] OR "trains"[All Fields]) OR ("social behavior"[MeSH Terms] OR ("social"[All Fields] AND "behavior"[All Fields]) OR "social behavior"[All Fields] OR "sociality"[All Fields] OR "social"[All Fields] OR "socialisation"[All Fields] OR "socialization"[MeSH Terms] OR "socialization"[All Fields] OR "socialise"[All Fields] OR "socialised"[All Fields] OR "socialising"[All Fields] OR "socialities"[All Fields] OR "socializations"[All Fields] OR "socialize"[All Fields] OR "socialized"[All Fields] OR "socializers"[All Fields] OR "socializes"[All Fields] OR "socializing"[All Fields] OR "socially"[All Fields] OR "socials"[All Fields]) OR "community based strategy"[All Fields] OR "community based intervention"[All Fields] OR "community intervention"[All Fields])) AND ((((((((((("COVID 19") OR (COVID)) OR ("Corona virus")) OR (tuberculosis)) OR (TB)) OR (Hepatitis)) OR (diarrhoea)) OR (Typhoid)) OR (dengue)) OR (influenza)) OR (Pneumonia))) NOT (((survey) OR ("clinical trial")) OR ("drug treatment"))) AND ((evaluationstudy[Filter] OR journalarticle[Filter] OR randomizedcontrolledtrial[Filter]) AND (humans[Filter]) AND (2011/1/1:2021/9/1[pdat]) AND (english[Filter]) AND (alladult[Filter]))

**Search Strategy for Web of science and Scopus**

**Population:**

“emerging country” OR “developing country” OR “least developed countries” OR "less developed country" OR "lesser developed country" OR "under developed country" OR "middle income country" OR "low income country" OR "lower income country" OR “underserved country” OR “deprived country” OR “poor country” OR LMICs OR “Low and middle income countries” OR “Low income and middle income countries” OR Africa OR Asia OR Caribbean OR “West Indies” OR “South America” OR “Latin America” OR “Central America” OR "Atlantic Islands" OR "Commonwealth of Independent States" OR "Pacific Islands" OR "Indian Ocean Islands" OR "Eastern Europe"

"poverty areas" OR "poverty" AND "areas" OR "poverty areas" OR "slum" OR "informal settlement" OR "informal settlements" OR "poverty" OR "poverty" OR "poor" OR "disadvantage*"

**Intervention:**

"prevent*" OR "controling" OR "controllability" OR "controllable" OR "controllably" OR "controller" OR "controller s" OR "controllers" OR "controlling" OR "controls" OR "prevention and control" OR "prevention" AND "control" OR "prevention and control" OR "control" OR "control groups" OR "control" AND "groups" OR "control groups" OR "communit*" OR "knowledge" OR "knowledge" OR "knowledge s" OR "knowledgeability" OR "knowledgeable" OR "knowledgeably" OR "knowledges" OR "attitude" OR "attitude" OR "attitudes" OR "attitude s" OR "practicability" OR "practicable" OR "practical" OR "practicalities" OR "practicality" OR "practically" OR "practicals" OR "practice" OR "practice s" OR "practiced" OR "practices" OR "practicing" OR "awareness" OR "awareness" OR "aware" OR "awarenesses" OR "mobils*" OR "mobiliz*" OR "engage*" OR "empower*" OR "capacity building" OR "education" OR "education" OR "training" OR "education" OR "train" OR "train s" OR "trained" OR "training s" OR "trainings" OR "trains" OR "social behavior" OR "social" AND "behavior" OR "social behavior" OR "sociality" OR "social" OR "socialisation" OR "socialization" OR "socialization" OR "socialise" OR "socialised" OR "socialising" OR "socialities" OR "socializations" OR "socialize" OR "socialized" OR "socializers" OR "socializes" OR "socializing" OR "socially" OR "socials" OR "community based strategy" OR "community based intervention" OR "community intervention"

**Outcome:**

"COVID 19" OR ("sars cov 2" OR "sars cov 2" OR "covid" OR "COVID 19" OR "COVID 19" OR "Corona virus" OR "tuberculosi" OR "tuberculosis" OR "tuberculosis" OR "tuberculoses" OR "tuberculosis s" OR "TB" OR "hepatitis" OR "hepatitis" OR "hepatitides" OR "hepatitis a" OR "hepatitis a" OR "diarrhea" OR "diarrhea" OR "diarrheas" OR "diarrhoea" OR "diarrhoeas" OR "typhoid fever" OR "typhoid" AND "fever" OR "typhoid fever" OR "typhoid" OR "typhoidal" OR "dengue" OR "dengue" OR "dengue s" OR "influenza s" OR "influenza, human" OR "influenza" AND "human" OR "human influenza" OR "influenza" OR "influenzae" OR "influenzas" OR "pneumonia" OR "pneumonia" OR "pneumoniae" OR "pneumonias" OR "pneumoniae s"
